# Game-related assessments for personnel selection: A systematic review

**DOI:** 10.3389/fpsyg.2022.952002

**Published:** 2022-09-28

**Authors:** Pedro J. Ramos-Villagrasa, Elena Fernández-del-Río, Ángel Castro

**Affiliations:** ^1^Department of Psychology and Sociology, Universidad de Zaragoza, Zaragoza, Spain; ^2^Department of Psychology and Sociology, Universidad de Zaragoza, Teruel, Spain

**Keywords:** personnel selection, gamification, serious games, job performance, applicant reactions, game-based assessment

## Abstract

Industrial development in recent decades has led to using information and communication technologies (ICT) to support personnel selection processes. One of the most notable examples is game-related assessments (GRA), supposedly as accurate as conventional tests but which generate better applicant reactions and reduce the likelihood of adverse impact and faking. However, such claims still lack scientific support. Given practitioners’ increasing use of GRA, this article reviews the scientific literature on gamification applied to personnel selection to determine whether the current state of the art supports their use in professional practice and identify specific aspects on which future research should focus. Following the PRISMA model, a search was carried out in the *Web of Science* and *Scopus* databases, identifying 34 valid articles, of which 85.3% are empirical studies that analyze five areas: (1) validity; (2) applicant reactions; (3) design of GRA; (4) personal characteristics and GRA; and (5) adverse impact and faking. Together, these studies show that GRA can be used in personnel selection but that the supposed advantages of GRA over conventional tests are fewer than imagined. The results also suggest several aspects on which research should focus (e.g., construct validity, differences depending on the type of game, prediction of different job performance dimensions), which could help define the situations in which the use of GRA may be recommended.

## Introduction

The industrial development of recent decades has led to the emergence of *digital selection procedures*, that is, any use of Information and Communication Technologies (ICT) to improve the personnel selection process (Woods et al., 2020). The incorporation of technology into selection has been remarkably successful, but research on this topic is still very scarce compared to its rapid adoption by professionals (Chamorro-Premuzic et al., 2017; Nikolaou, 2021).

Digital selection procedures go beyond a mere change to technology-based assessment (e.g., face-to-face interview vs. interview by videoconference). Instead, they may involve changes in the assessment formats, the evaluation of work-performance predictors, and test correction (Tippins, 2015). One of the most noteworthy examples is gamification and game-related assessments (GRA; Woods et al., 2020).

Gamification consists of incorporating game elements into nongaming contexts (Nacke and Deterding, 2017), whereas GRA are assessments based on gamification. Interest in GRA for personnel psychology is now greater than ever: we have the recent reviews on technology applied to human resources in which this technique has its own section (cfr. Tippins, 2015; Woods et al., 2020; Nikolaou, 2021); the most recent conferences of SIOP and EAWOP includes four and three presentations about games and personnel selection, respectively; and the last volume of *International Journal of Selection and Assessment* publishes a special issue dedicated to this topic. Although GRA seem to be inextricably linked to technology (e.g., Tippins, 2015; Landers and Sanchez, 2022), game-related evaluations that do not require the use of ICT can be designed and applied (Melchers and Basch, 2022), for example, escape rooms, which can be developed without ICT (e.g., Connelly et al., 2018). However, successful worldwide games for personnel selection are technology-based (e.g., Nawaiam, Owiwi, Wasabi Waiter), and until now, research has been practically based only on them. Hence, this article also focuses on technology-based GRA.

The use of GRA in personnel selection is growing because they appear to reduce the risk of faking and improve candidates’ reactions without a substantial loss of predictive validity (Melchers and Basch, 2022; Wu et al., 2022). This systematic review article was born within this context. However, the increasing use of GRA by personnel selection professionals does not necessarily imply that their use is recommended. We need scientific evidence to support the equivalence of GRA to conventional selection methods and determine whether they provide added value (Nikolaou et al., 2019). This issue is relevant because the selection processes must meet psychometric requirements and comply with the legality and the promotion of applicants’ positive reactions (Salgado et al., 2017). Therefore, we propose the present systematic review to determine the possible favorable evidence for GRA use in professional practice and to analyze different types of GRA to guide future research.

### Game-related assessments: Concepts and classification

GRA are based on games. What elements characterize a game? Following Landers et al. (2018), they are the constructs that make up the play experience under different taxonomies. Bedwell et al.’s (2012) taxonomy is one of the most accepted in the organizational field, establishing nine categories described in Table 1.

**Table 1:** Taxonomy of elements that make up the gaming experience.

Category
1. *Action language*. How the interaction between person and machine occurs (e.g., pointing and pressing, scrolling with the keys).
2. *Assessment*. How game information and goal achievement are recorded (e.g., scores, progress bars).
3. *Conflict / Challenge*. Difficulty of the game, the type of problems that players must face, and the degree of uncertainty or surprise when encountering such problems.
4. *Control*. Variety of actions that the player can deploy (i.e., agency).
5. *Environment*. The place where the action of the game occurs and the player is situated.
6. *Game fiction*. Degree of realism, whether the player is knowledgeable about the game world and whether the player’s actions within the game are represented directly or indirectly.
7. *Human interaction*. Whether there is interaction between players and what type (e.g., comparative ranking, player vs. player matches).
8. *Immersion*. To what extent the game contains perceptual elements that encourage the player to immerse themselves in the game.
9. *Rules/Goals*. The game has clear rules known to the player.

Adapted from Bedwell et al.’s (2012).

From a theoretical point of view, GRA are applications of gamification science, “a social scientific, post-positivist subdiscipline of game science that explores the various design techniques and related concerns that can be used to add game elements to existing real-world processes” (Landers et al., 2018, p. 318). An example is the work setting (Armstrong and Landers, 2018) and its processes, such as recruitment (Korn et al., 2018), selection (Hommel et al., 2022), or training (Armstrong and Landers, 2017).

However, gamification does not reflect the different approaches to the relationship between play and human resources, as it can generate confusion between researchers and personnel selection professionals. To avoid this, Landers and Sanchez (2022) have proposed differentiating three terms: *game-based assessment*, *gameful design assessment*, and *gamification assessment*. Game-based assessment refers to an evaluation method, while the other two terms refer to the strategy used when designing evaluation tests. We will define each of them following these authors’ proposal, qualifying it when necessary to establish a complete taxonomy.

Game-based assessment refers to a selection method, that is, it measures a wide range of job-related constructs through games (Wu et al., 2022). Within game-based assessment, we could also differentiate between theory-driven games, designed to evaluate constructs that are related to job performance, and data-driven games, where game scores are related to the criterion instead of the constructs’ psychological entity (Landers et al., 2021; Auer et al., 2022). An example of game-based assessment is Virus Slayer (Wiernik et al., 2022), a serious game to assess candidates for cyber occupations in the United States Air Force (USAF).

Gameful design assessment consists of using game elements to design a new assessment, as in the case of Owiwi, a situational judgment test to evaluate professional skills to which game elements have been added, such as the choice of a character, a narrative, etc. (Georgiou et al., 2019).

Gamification assessment is a redesign strategy based on an existing assessment test to which game elements are added, modifying it in some way. An example of this strategy is Hommel et al.’s (2022) modification of the Wisconsin Card Sorting Test by incorporating a narrative context, the possibility of earning points, and a progression graph during the game.

Landers and Sanchez (2022) focus on games developed to evaluate what other classifications have called *serious games* (Wiernik et al., 2022). However, conceptually, we can also include the possibility of using conventional games to gather information about specific abilities, such as general cognitive ability (Quiroga et al., 2019; Peters et al., 2021). Therefore, we propose to call this second type of GRA *playful games*.

**Figure 1:**
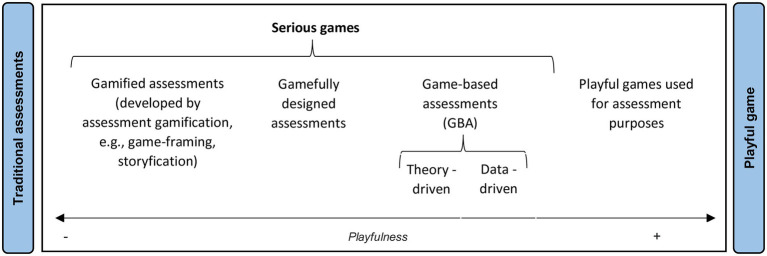
A classification of game-related assessments.

From Landers and Sanchez’s (2022) terms, we propose a classification of GRA. As shown in Figure 1, the classification begins on a continuum: at one end are the traditional assessments (e.g., tests, simulations), and at the other end, the playful games created for fun. Thus, we distinguish at least four types of GRA: (1) gamified assessment (e.g., Hommel et al., 2022); (2) gamefully designed assessment (e.g., Georgiou, 2021); (3) game-based assessments (e.g., Wiernik et al., 2022); and (4) playful games used for assessment purposes (e.g., Sanchez et al., 2022).

It has been hypothesized that if the assessment test is presented as a game (i.e., the closer it is to the playfulness extreme in Figure 1), the applicants’ motivation will increase (Coovert et al., 2020), their propensity to fake will decrease, as will their tendency to offer a better self-image, because they are encouraged to engage in the game (Landers and Sanchez, 2022). Moreover, the game will elicit better reactions from the applicants, such as those referring to organizational attractiveness (Gkorezis et al., 2021). For all the above, this classification is functional.

### Requirements for the use of game-related assessments in personnel selection

Although GRA are growing among professionals, we must be cautious when recommending its use. Therefore, research should provide empirical evidence to support the rigor of GRA, considering the influence of the type of GRA on the results (Chamorro-Premuzic et al., 2017; Landers and Sanchez, 2022).

Concerning the rigor of the assessments, GRA used for personnel selection must meet psychometric standards (Salgado et al., 2017; Landers et al., 2021; Wiernik et al., 2022): (1) acceptable reliability to ensure consistency in the measure; (2) construct validity, verifying that GRA measure what is meant to be measured; (3) predictive validity, to predict the criterion (e.g., job performance); (4) freedom from bias, so that applicants’ scores are not influenced by their personal characteristics (e.g., sex, experience with video games) or, if this occurs, knowing how to correct this effect when estimating the scores. Moreover, GRA should promote positive applicant reactions.

The influence of GRA characteristics on assessment results must yet be explored because the use of GRA in selection is still in its infancy (Landers and Sanchez, 2022). However, there are enough studies to evaluate them concurrently and identify which issues future GRA research will need to address.

Bearing in mind both issues, rigor in the evaluation and the particular characteristics of each GRA, we propose to review the developing scientific literature on GRA applied to personnel selection with two objectives: (1) to determine whether the current state of the art supports their use in professional practice; (2) to identify specific aspects on which future research should focus. This will mitigate the general public’s misgivings concerning this new form of evaluation (al-Qallawi and Raghavan, 2022) and, at the same time, it will help to clarify the incipient research on games-related assessment, which so far has shown some inconsistency, for example with the use of terms (Landers and Sanchez, 2022).

## Materials and methods

### Inclusion criteria

Three inclusion criteria were established before conducting the review: (1) we would accept only published papers; (2) written only in English or Spanish; and (3) focused on technology-based GRA for personnel selection. There were no restrictions on participants’ populations, geographical or cultural origin, research design, or period in which studies were published.

### Literature search

We followed the PRISMA statement for this review (Page et al., 2021) and the guidelines based on MARS developed by Schalken and Rietbergen (2017), using Web of Science (WoS) and Scopus as databases. The keywords used were [“personnel selection”] and [“gamification” OR “gamified” OR “serious game” OR “game”] in the field “topics” in WoS, and as “Title, Abstract, and Keywords” in Scopus. The search was performed in March 2022. A total of 105 results were found in WoS and Scopus. Following journal guidelines on systematic reviews, we only considered published studies.

**Figure 2:**
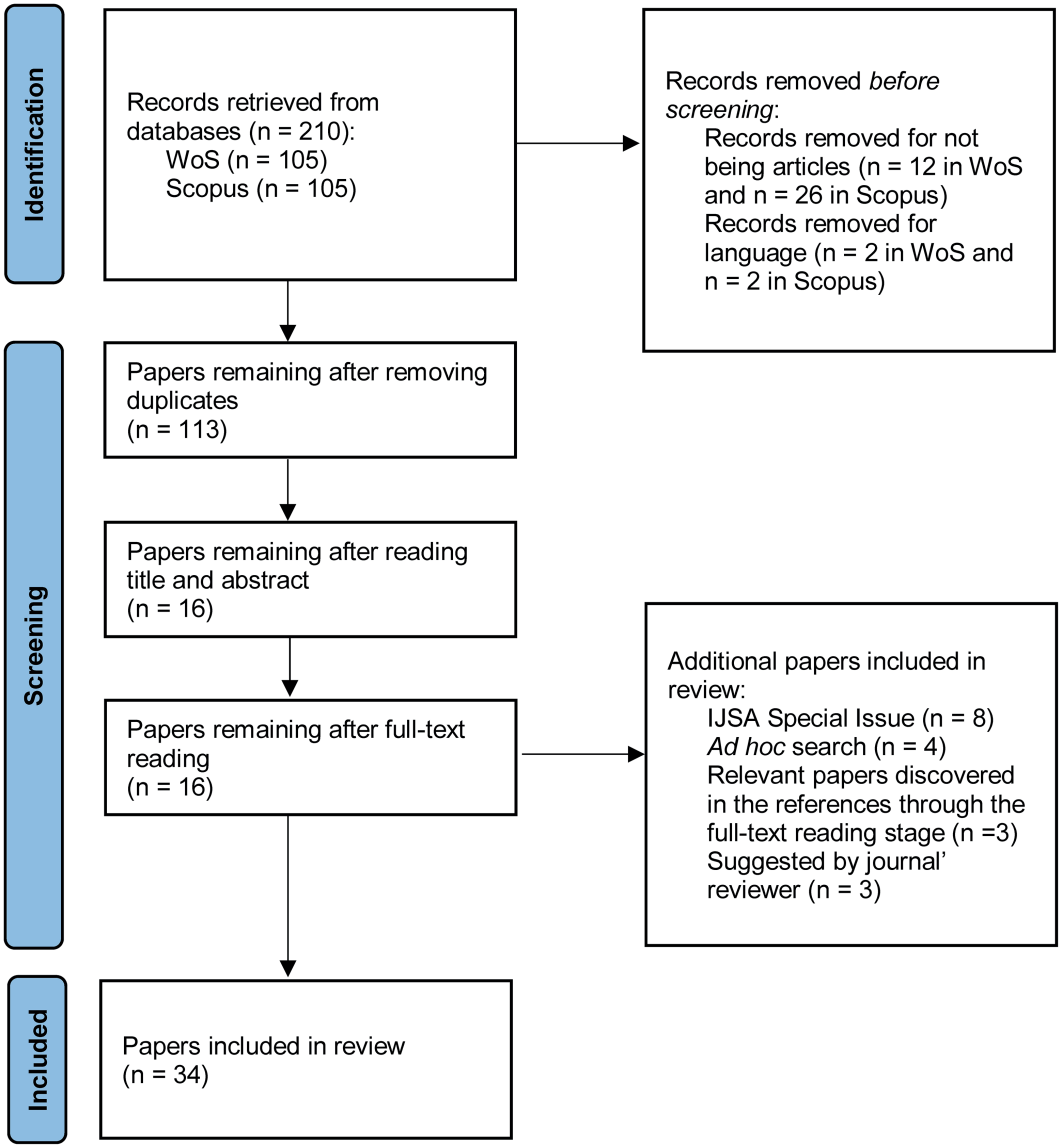
PRISMA flow diagram for the systematic review.

After removing duplicates, 113 articles remained. Screening and coding were performed by the first author, whose qualification is a Ph.D. in Work and Organizational Psychology. Screening was based on title and abstract and provided 16 suitable articles according to our inclusion criteria. After reading the whole article, one was removed (i.e., Connelly et al., 2018) because it was not related to technology-based GRA. The remaining papers were included in the final analysis. Articles from three additional sources were also incorporated: (1) eight papers from the special issue on gamification applied to personnel selection from the *International Journal of Selection and Assessment*, published in March 2022 (some papers from the special issue were already included in the database search, the remaining were published after the search had been performed); (2) four papers known to the authors of this review which were not detected by the search due their title, abstract, or keywords; (3) three relevant papers discovered in the references at the full-text reading stage; and (4) three articles suggested by one of the journal reviewers, two that were not found in the database search, and one that was ahead-of-print after the search was performed. Thus, the final number of articles was 34 (see Figure 2 for a diagram describing the whole process and Supplementary Material 2 for the list of articles included). All articles were written in English except for one in Spanish (i.e., Albadán et al., 2016). One of the articles was obtained by contact with the correspondence author.

## Results

As a first approximation, the 34 articles identified in the search were classified as theoretical or empirical. In empirical articles, the type of GRA analyzed was identified according to the classification presented in Figure 1. As shown in Table 2, most of the research was empirical (85.3%) and was carried out based on the four types of GRA identified. Most articles dealt with gamified assessments (29.4% of the total articles included in the review). Interest in GRA has been growing in recent years, with one article published both in 2012 and 2018, two published in 2016 and 2017, three in 2019, five in 2020, six in 2021, and fourteen in 2022 (year of publication of the special issue of *The International Journal of Selection and Assessment*).

**Table 2:** Classification of articles identified in the systematic review.

Type of article	Type of GRA	Reference
Theoretical	Not applicable	Armstrong et al. (2016) Küpper et al. (2021) Landers and Sanchez (2022) Nikolaou (2021) Woods et al. (2020)
Empirical	Gamified assessment	Collmus and Landers (2019) Hilliard et al. (2022) Hommel et al. (2022) Buil et al. (2020) Ellison et al. (2020) Harman and Brown (2022) Landers and Collmus (2022) Landers et al. (2020) Laumer et al. (2012) McChesney et al. (2022)
	Gamefully designed assessment	Brown et al. (2022) Georgiou (2021) Georgiou et al. (2019) Georgiou and Lievens (2022) Georgiou and Nikolaou (2020) Gkorezis et al. (2021) Nikolaou et al. (2019)
	Game-based assessment theory-driven	Albadán et al. (2016) Auer et al. (2022) Landers et al. (2021) Wiernik et al. (2022) Wu et al. (2022)
	Unknown	Egol et al. (2017) Melchers and Basch (2022)
	Playful game	Sanchez et al. (2022)
	Other/Not applicable	Albadán et al. (2018) al-Qallawi and Raghavan (2022) Balcerak and Woźniak (2021) Formica et al. (2017)

### Theoretical articles

Concerning the theoretical articles, Armstrong et al. (2016) are the first to delimit GRA. According to them, GRA are more than just digital versions of situational judgment tests. They outline the need to establish Industrial-Organizational Psychology literature on gamification and its increase among practitioners:

Gamification of assessment will not disappear from practice, just as people will not stop using the Internet, mobile devices, or video-based interviews [...] By first understanding gamification, I-Os can then apply theory to gamification in order to improve applicant and employee assessment in ways that matter to firms and test takers. (p. 676)

Of the remaining theoretical articles, two were reviews on the role of ICTs for personnel selection (i.e., Woods et al., 2020; Nikolaou, 2021). In both of them, GRA are currently presented as one of the areas of greatest interest due to the rise of digital selection procedures. We highlight GRA’s potential advantages (better psychometric characteristics and applicant reactions, less faking, social desirability, and bias) and the existence of emerging studies.

The article of Küpper et al. (2021) has a different goal: they propose a conceptual framework that explains how serious games can be used for employer branding purposes, a reasonable goal given the alleged relationship between GRA and applicant reactions. Their framework considers game-specific factors (e.g., game genre, level of realism), player-specific factors (e.g., self-perceived innovativeness, prior application experience), and learning (cognitive and affective) as antecedents of three types of employer branding outcomes. Although suggestive, the model requires empirical validation.

These articles are an excellent introduction to the article by Landers and Sanchez (2022), prepared as an editorial for the aforementioned special issue in *The International Journal of Selection and Assessment*. They present the articles included in the issue, clarify the above-mentioned concepts (i.e., game-based assessment, gameful design assessment, gamification assessment), explain the *core gameplay loop,* which helps understand the gaming experience, and provide some guidelines for the design of game-based assessments. They also propose explanatory models of how GRA influences applicant reactions and reduces faking behavior. However, as with the model of Küpper et al. (2021), empirical validation is still necessary.

### Empirical articles

Most of the empirical articles are cross-sectional and use student samples. Specific information about each article (type of design, sample, GRA used, constructs evaluated) is presented in Supplementary Material 3. We classified their findings into five areas: (1) validity; (2) applicant reactions; (3) design of GRA; (4) personal characteristics and GRA; and (5) adverse impact and faking. Next, we will discuss each of these categories in detail. A summary of the findings by category is presented in Table 3. Reference to GRA types follows the classification shown in Figure 1.

**Table 3:** Main areas and findings of empirical research on GRA.

Area	Results	References
1a. Construct validity	1. Studies on construct validity GRA show inconclusive results. Game design seems to have a significant influence on validity.	Auer et al. (2022) Brown et al. (2022) Georgiou et al. (2019) Harman and Brown (2022) Hilliard et al. (2022) Landers and Collmus (2022) Sanchez et al. (2022) Wu et al. (2022)
	2. Most studies on construct validity are on GRA that measure personality. However, better results have been obtained by evaluating cognitive ability and competences.	Auer et al. (2022) Georgiou et al. (2019) Harman and Brown (2022) Hilliard et al. (2022) Landers and Collmus (2022) Landers et al. (2020) Wu et al. (2022)
1b. Predictive validity	1. GRA can predict the criterion. The evidence has focused on academic performance and task performance.	Auer et al. (2022) Egol et al. (2017) Hommel et al. (2022) Landers et al. (2021) Melchers and Basch (2022) Nikolaou et al. (2019)
	2. There is evidence of incremental validity of GRA over traditional tests.	Nikolaou et al. (2019) Landers et al. (2021)
1c. Discriminant validity	1. The sole study shows that the GRA analyzed has discriminant validity.	Wiernik et al. (2022)
2. Applicant reactions	1. GRA promote positive reactions in the applicants, especially concerning organizational attractiveness.	al-Qallawi and Raghavan (2022) Balcerak and Woźniak (2021) Collmus and Landers (2019) Georgiou (2021) Georgiou and Lievens (2022) Georgiou and Nikolaou (2020) Gkorezis et al. (2021) Harman and Brown (2022) Hommel et al. (2022) Landers et al. (2021) Landers and Collmus (2022)
	2. The perceived organizational attractiveness of being assessed with GRAs is due, at least in part, to the effect that the enjoyment and flow of the game has on the applicant’s perception of how innovative and competent the organization is.	Georgiou and Lievens (2022)
	3. Negative reactions to GRA are usually related to specific aspects of technology (bugs, connection errors, etc.) and not to the content of the test itself.	al-Qallawi and Raghavan (2022)
	4. Indicating that a test is a game (game-framing), even when it is not, improves the applicant’s reactions to the test.	Collmus and Landers (2019) McChesney et al. (2022)
	5. GRA are usually better valued than conventional selection methods, except in the case of job-relatedness. The magnitude of this increase does not seem to be very high, and there are cultural differences.	Balcerak and Woźniak (2021) Collmus and Landers (2019) Georgiou and Nikolaou (2020) Georgiou (2021) Harman and Brown (2022) Hommel et al. (2022) Landers and Collmus (2022) Landers et al. (2020, 2021)
	6. Providing explanations to applicants before the application of GRA it is advisable to increase its positive reactions.	Georgiou (2021)
	7. Some personal and GRA characteristics have a positive impact on applicant reactions: being male, having experience playing video games, having high self-efficacy for technology, utility, equity, and perceived fun, and the perception of ease of use.	Buil et al. (2020) Ellison et al. (2020) Georgiou and Nikolaou (2020) Gkorezis et al. (2021) Laumer et al. (2012) McChesney et al. (2022)
3. Design of GRA	1. It is possible to design theory-driven GRA.	Landers et al. (2021)
	2. It is preferable to use GRA developed for evaluation purposes than playful games.	Sanchez et al. (2022)
	3. The use of virtual reality is only recommended when it adds value to the evaluation.	Sanchez et al. (2022)
	4. Considerable data are generated during the game that can be analyzed in various ways. Using this data to make assessments with multiple predictors improves GRA outcomes as an assessment test. Estimating reliability by means of test–retest is recommended in these cases.	Auer et al. (2022) Albadán et al. (2016, 2018) Wiernik et al. (2022) Sanchez et al. (2022) Wu et al. (2022)
	
4. Personal characteristics	1. Although it is thought that being men and young is associated with better outcomes in GRA, studies confirming this difference often find modest results that are probably irrelevant in a real context.	Melchers and Basch (2022)
	2. Education, experience with computers, and self-efficacy for video games may influence GRA scores. In addition, people who regularly play video games have greater emotional stability.	Formica et al. (2017) Hommel et al. (2022) Wiernik et al. (2022) Sanchez et al. (2022)
	3. There is a relationship between the Big Five and the scores of the GRA, but the specific relationship varies depending on the type of game.	Sanchez et al. (2022) Wu et al. (2022)
5. Adverse impact and faking	1. At the very least, GRA have no more adverse impact than a conventional test. Some studies find more positive results. The only study that analyzes faking reports better results than the original conventional test.	Brown et al. (2022) Hilliard et al. (2022) Landers et al. (2021) Landers and Collmus (2022)

#### Validity

Research on the validity of GRA has fundamentally addressed two issues, construct validity and predictive validity, although we also found one study on discriminant validity.

##### Construct validity

Most of the articles on construct validity focus on personality, although analyzing very diverse issues. First, Hilliard et al. (2022) design a test to evaluate the Big Five based on items in which the applicant must choose the image they think best describes them. The results show good levels of convergent validity with a conventional personality questionnaire. The test elaborated through storyfication by Landers and Collmus (2022) also gives samples of construct validity. However, the authors acknowledge that modifying the original test has turned the gamified version into a different instrument, with its advantages and disadvantages, concluding that this must be considered when gamifying a test through storyfication. On the other hand, Harman and Brown (2022) examine whether including evocative images in a text-based game (i.e., McCord et al., 2019) will improve its construct validity. Their results show that, as with the original version, the associations with the Big Five measured with a conventional test are modest, and there are no significant differences between the two versions of the game. We also include herein the work of Sanchez et al. (2022), who use playful games to evaluate sensation-seeking, height aversion, and risk-taking, only finding a relationship between the game scores when evaluating the first two constructs and openness to experience. The latest study on construct validity, specifically on personality, offers unfavorable results: Wu et al. (2022) develop two game-based assessments to evaluate facets of conscientiousness but which really assess cognitive ability and the remaining Big Five. To their surprise, they find that the games they used actually measure cognitive ability (verbal ability and matrix reasoning) better than the Big Five. In conclusion, they recommend that game-based assessments be designed to consider the possible contamination of the information collected, as occurs with situational judgment tests and assessment centers.

The rest of the articles on construct validity analyze cognitive ability, competences, and emotional intelligence. Concerning cognitive ability, Auer et al. (2022) investigate whether the large amount of data generated by playing a game (i.e., trace data modeling) can predict cognitive ability and conscientiousness and whether these data have an incremental value compared to using only the score generated by the game for prediction. Their results show that trace data modeling predicts cognitive ability but not conscientiousness, and they delve into the difficulties encountered in assessing personality with game-based assessments.

Concerning competences, Georgiou et al. (2019) analyzed the construct validity of Owiwi, a gamefully designed assessment for the evaluation of key competences in the workplace (i.e., resilience, flexibility, adaptability, and decision-making). The authors carry out two studies, the first to develop the scenarios that will be part of the test with the collaboration of 20 human resources experts, and the second to validate the test in a sample of 321 university students and replicate it in a sample of 410 workers and people in the process of job-seeking. Their results indicate that Owiwi shows adequate content validity. Landers et al. (2020) also research competences, showing that the inclusion of game elements in a situational judgment test (control, immersion, interaction) does not substantially affect the construct validity.

As for emotional intelligence, the two existing pieces of research show limited support. Brown et al. (2022) propose a gamefully designed assessment in which the social interactions that make up the items are performed by abstract shapes. The game scores show a moderate association with a situational judgment test. Sanchez et al. (2022) examine whether a playful game of virtual reality can be used to evaluate emotional intelligence, finding a moderate relationship with a conventional test. In fact, the associations with measures of personality turned out to be higher than the associations with emotional intelligence.

Taken together, the research on the construct validity of GRA shows inconclusive results, underscoring the importance of game design to evaluate adequately what one intends to evaluate.

##### Predictive validity

While research on construct validity has yielded mixed results, research on predictive validity is more promising. Thus, using samples composed totally or mainly of students, a relationship has been found between various GRA (i.e., Cognify, Owiwi, Wasabi Waiter, and Wisconsin Card Sorting Test) and academic performance (Egol et al., 2017; Nikolaou et al., 2019; Landers et al., 2021; Auer et al., 2022; Hommel et al., 2022). However, Landers and Collmus’ (2022) attempt to gamify a personality measure through storyfication does not find a relationship with the grade point average. Focusing on task performance, two of the above-mentioned works include small samples of workers where this relationship with self-reported job performance is found (Nikolaou et al., 2019) and, in another case, with supervisory ratings (Landers et al., 2021). Also, Melchers and Basch (2022) find positive associations between the scores of more than one thousand applicants in a business simulation GRA and job-related performance in an assessment center.

As GRA seem to show predictive validity, the next question is whether they show incremental validity compared to traditional tests. In this sense, Nikolaou et al. (2019) find that if cognitive ability and personality are evaluated, the GRA to evaluate competences (i.e., Owiwi) only predict academic performance. Landers et al. (2021) obtained a similar result, finding that Cognify, a game to evaluate cognitive ability, has a cumulative effect on the prediction of academic performance if a cognitive ability test is added to the game score, but not vice versa.

##### Discriminant validity

Wiernik et al.’s (2022) study is the only one on this type of validity. Their article documents the creation of the game-based assessment Virus Slayer. This game evaluates six competences relevant to the USAF: analytical thinking, active learning, deductive reasoning, systems thinking, adaptability, and situational awareness. Their results show that the game has an adequate discriminant validity, and it can be improved by estimating the scores with three different types of information: multiple gameplay phases, diverse game behavioral indicators, and residualizing game behavioral indicators.

#### Applicant reactions

Undoubtedly, most research has focused on the category of applicant reactions. In fact, more than half of the empirical research on GRA deals with this issue. Thus, we can confirm that GRA promote positive reactions in the applicants (Georgiou, 2021; al-Qallawi and Raghavan, 2022; Georgiou and Lievens, 2022) and tend to be better valued than conventional selection methods (Collmus and Landers, 2019; Georgiou and Nikolaou, 2020; Landers et al., 2021; Harman and Brown, 2022; Hommel et al., 2022). It is noteworthy that this result seems consistent across the different types of GRA, as these investigations have been conducted with very diverse games, starting with traditional assessment and going on to include serious games.

Delving into these investigations, we can qualify this general idea. Firstly, we recognize the importance of framing: the mere fact of defining an online evaluation as a game improves the applicants’ reactions, as they consider the organization to be more innovative and attractive (McChesney et al., 2022) and the test to be shorter (Collmus and Landers, 2019).

On the other hand, we must acknowledge that not all reactions are positive. Thus, al-Qallawi and Raghavan (2022) study the reactions generated by nine serious games published in mobile application stores (App Store and Google Play). Through a qualitative approach, using natural language processing, they identify a general tendency to value GRA positively. Negative reactions are due to specific technology-related aspects and not to the evaluation itself, such as the presence of bugs or the game’s design. In addition, they find that negative reviews are often made by people who distrust GRA as an evaluation method. In contrast, Landers and Collmus (2022) evaluate the reactions to a conventional personality measure and a gamified one by introducing a narrative (*storyfication*), finding better results for the GRA, except for face validity.

Another necessary qualification is that not all types of applicant reactions (e.g., fairness, satisfaction) are valued equally. Organizational attractiveness appears to experience the most significant growth (Georgiou and Nikolaou, 2020; Gkorezis et al., 2021). However, in other investigations with the same gamefully designed assessment, Georgiou (2021) finds that the game is perceived as less job-related than its conventional counterparts. In another study mentioned in the same article, Georgiou shows that providing explanations to applicants has a more positive effect on their reactions to GRA than to conventional tests. In any event, two studies by Landers et al. (2020, 2021) find that, compared with conventional tests, the gain of GRA is minimal, inviting us to reflect on the magnitude of the improvement involved when using GRA. In addition, as perceptions of justice are subject to cultural differences (Anderson et al., 2010), it is also interesting to value research conducted outside the Anglo-Saxon context. In this regard, Balcerak and Woźniak (2021) conduct a study in Poland where they analyze the reactions to different traditional selection methods compared to modern and technology-based ones. Unlike the rest of the research, their results show a clear preference for traditional methods, although it is noteworthy that, of the new methods, after the e-interview, GRA are the best valued.

The relationship between personal characteristics and GRA in applicant reactions has also been the subject of research. Ellison et al. (2020) find that being male, having high self-efficacy beliefs for technology, and perceived fairness influence reactions. Along the same lines, Gkorezis et al. (2021) find that GRA make the company seem more attractive, but only if the participants have previous experience playing video games. On the other hand, Buil et al. (2020) propose a theoretical model in which personal characteristics (i.e., competence and autonomy in the use of ICT) influence intrinsic motivation, and this, in turn, influences applicant reactions. Their results support the proposed model, finding relationships for all the variables except for the relationship between intrinsic motivation and perceived usefulness. In contrast, the openness to experience trait does not influence the attractiveness of GRA for candidates (Georgiou and Nikolaou, 2020; McChesney et al., 2022). Finally, Laumer et al. (2012) propose the possibility of using GRA as a tool for candidates’ self-evaluation to decide whether to apply for a job. Using a sample of 1882 job-seekers, they find that the decision to resort to this game for self-assessment is based on the perception of: (1) ease of use; (2) utility; (3) fun; and (4) fairness in the selection process. It is noteworthy that they do not find any influence of the perception of privacy although this issue has repeatedly worried researchers (Tippins, 2015).

The underlying mechanisms by which GRA exerts a positive effect on applicant reactions have recently begun to be explored. Using a longitudinal study and an experiment, Georgiou and Lievens (2022) find that the enjoyment and flow of GRAs caused applicants to perceive the organization as more innovative and competent, and, consequently, more attractive.

#### Design of game-related assessments

The design of GRA has also been of interest to researchers, although with less emphasis and much more diverse studies.

Firstly, we highlight the work of Landers et al. (2021), who explain and illustrate how to design theory-driven game-based assessments based on research on game design and psychometrics. This is a good guide for future researchers and practitioners. According to these authors, serious games developers may use the design thinking theory taken from human-computer interaction literature. Design thinking proposes five stages for the development of game-based assessment that may be iterated until the final version of the game is reached: (1) *empathizing*, in which the constructs to be evaluated are identified (e.g., using job analysis); (2) *definition*, in which the actual application context of game is defined, and the developers try to solve technical problems (e.g., supported devices, minimum requirements); (3) *ideating*, in which the assessment and the technical team build a shared mental model to develop a useful prototype; (4) *prototyping*, in which the teams create the planned product for trial, either in the form of a low- or a high-fidelity prototype; and (5) *testing*, in which they assess the degree to which the game meets the pre-established goals (e.g., reliability, validity, reactions).

On the other hand, the study of Sanchez et al. (2022) is the only one that focuses on the use of playful games for selection, in particular, commercial virtual reality video games to evaluate performance-related constructs (e.g., emotional intelligence, risk-taking). They find very limited support for the use of these GRA, concluding that it is better to use tests designed specifically for evaluation purposes. In the particular case of virtual reality, they recommend only using it when its particularities offer some advantage to the evaluation that cannot be obtained by other means.

Another issue related to the design of GRA is the possibility of taking advantage of the data generated while playing. In this sense, as already mentioned regarding validity, Auer et al. (2022) show the options of trace data modeling to evaluate different predictors and their relationship with the criterion. As far as design is concerned, they find that using this additional information improves the prediction compared to using the game score exclusively. They also highlight in their conclusions the importance of the design phase of the GRA, clearly defining the constructs that one wants to evaluate. The contributions of Albadán et al. (2016, 2018) align with this issue. In 2016, they propose a video game to select senior management personnel. The game consists of managing a herd of animals and facing different random events. Although their design is theory-driven, they do not elaborate their proposal very much or provide evidence of the reliability or validity of the GRA. For their part, Wiernik et al. (2022) achieve similar results with Virus Slayer. Their research suggests that a multifactorial approach, employing different types of information generated by the game, can lead to better results. However, in the opinion of Sanchez et al. (2022), using these different measurement forms pose problems in estimating the reliability through internal consistency. As an alternative, they propose the estimation of reliability by test–retest, presenting adequate results and showing that it is a viable alternative for GRA that use this type of information.

The last issue related to the design of GRA is the treatment of the data collected through the game. Some research proposes strategies based on different analysis techniques. Thus, Albadán et al. (2018) show how fuzzy logic can help classify the applicants when information referring to their behavior is collected in the game. Moreover, this approach is not alien to personnel selection, as its application has already been proposed during the recruitment phase (i.e., García-Izquierdo et al., 2020). Instead, Auer et al. (2022) propose using machine learning, showing that, at least when predicting with GRA, it has incremental validity over traditional approaches. Wu et al. (2022) also propose using machine learning as an alternative to regression, especially in GRA that estimate more variables than the number of applicants evaluated. Finally, Wiernik et al. (2022) suggest that the information collected during the game can be estimated utilizing continuous-time latent growth curve models to improve the prediction.

#### Personal characteristics and game-related assessments

The influence of personal characteristics in the scores is relevant to any method used in selection. In the case of GRA, there exists a stereotype that being male and young is commonly associated with better performance in videogames (Fetzer et al., 2017). However, based on the conflicting results found in this review, this relationship is more inconsistent than thought (Ellison et al., 2020; Georgiou and Nikolaou, 2020; Balcerak and Woźniak, 2021; Gkorezis et al., 2021; Landers et al., 2021; Hommel et al., 2022; McChesney et al., 2022; Wiernik et al., 2022). Studies that find sex differences usually report poor effect sizes and are probably not very relevant in natural contexts (Melchers and Basch, 2022).

Other sociodemographic characteristics that are related to GRA scores are education (Wiernik et al., 2022) and computer experience (Hommel et al., 2022). As for the relationship with the use of video games, there is some evidence that self-efficacy in playing video games positively influences GRA scores, but the results are inconsistent (Sanchez et al., 2022). Playing experience, on the other hand, does not seem to affect evaluations with GRA (Hommel et al., 2022). Complementing these studies, Formica et al. (2017) find that people who play video games show greater emotional stability than those who do not, specifically, higher levels of emotional control and impulse control.

Regarding personality, it is also noteworthy that the relationship of the Big Five with GRA scores varies depending on the videogame (Sanchez et al., 2022; Wu et al., 2022).

#### Adverse impact and faking

The idea that GRA allow for unbiased assessments and prevent faking is probably one of the main arguments in their favor. Research on this is still developing and seems to support this idea, albeit with nuances. Concerning adverse impact, some studies find no difference in scores based on gender, race/ethnicity, or education when using GRA (Brown et al., 2022; Hilliard et al., 2022), but others, such as that of Landers et al. (2021), find similar results to conventional tests of adverse impact by race. These results may be related to the construct they evaluate because the first two papers focus on personality and that of Landers et al. on cognitive ability. Thus, in the absence of more research in this regard, we conclude that GRA have no more adverse impact than a conventional test.

With regard to faking, the only research that addresses this issue shows that a GRA made by means of storyfication is more resistant to faking than the original test (Landers and Collmus, 2022).

## Discussion

This systematic review article has focused on using GRA in personnel selection with two objectives: (1) to determine whether the current state of the art supports their use in professional practice; (2) to identify specific aspects on which future research development should focus. Next, we will address the two objectives from the information obtained through the systematic review.

### Using game-related assessments for personnel selection

As mentioned, the GRA use may be recommended if they show: (1) reliability; (2) construct validity; (3) predictive validity; (4) freedom from bias; and (5) positive applicant reactions. After reviewing the empirical research, we can conclude that, indeed, GRA can be used for personnel selection, taking into account some considerations.

First, the results on construct validity reveal inconsistent outcomes, and this should be improved overall. One possible avenue may be to focus on developing games through gamification assessment, rather than gameful design and game-based assessment, at least until research on game design identifies how to build tests closer to games without losing validity. Second, while GRA have been shown to predict academic performance and task performance, their results are not much better than the existing traditional tests. In fact, GRA seem to benefit from the complementary use of conventional testing, but not vice versa (Landers et al., 2021). Thus, in the absence of further research in this regard, we cannot consider that GRA has greater predictive validity than other methods. The results on personal characteristics, adverse impact, and faking invite optimism but are still too scarce to draw conclusions. These applicant reactions are possibly the aspect in which GRA obtain their best results, but the game-framing phenomenon (Collmus and Landers, 2019; McChesney et al., 2022) suggests that it may not be necessary to make great efforts for the development of GRA, but to know how to present test-type evaluations or simulations more attractively to applicants.

Considering all the above, and taking into account that research on this issue is still ongoing and that it is difficult to draw conclusions applicable to all GRA (Landers et al., 2021; Wu et al., 2022), we consider that they do not offer sufficient advantages to recommend their use over conventional methods unless it is thought that improving applicant reactions, especially organizational attractiveness (Georgiou and Nikolaou, 2020; Gkorezis et al., 2021), offers added value to the specific evaluation process. In any case, practitioners who wish to use GRA should only use games developed specifically for that purpose (Sanchez et al., 2022), based on some psychological theory (Landers et al., 2021), and offering adequate psychometric characteristics. In this sense, the GRA with the most empirical support so far is Owiwi (cfr., Georgiou et al., 2019; Nikolaou et al., 2019; Georgiou and Nikolaou, 2020; Gkorezis et al., 2021), although it suffers from a lack of research with more samples of workers and applicants. In addition, the game has been expanded to evaluate new competences but to date, no research has been published to support its use.

All of these statements, however, are subject to future verification. The present review has also shown that, given the breadth of GRA types, the different constructs to be evaluated, and the ways of collecting and treating data, we really know very little. Fortunately, it has also allowed identifying concrete demands for future research. That is what we will deal with next, answering our second research question.

### Avenues for further research

Undoubtedly, the main recommendation for the future is to contextualize research on GRA by drawing on existing taxonomies, for example, classifying the game according to the categorization proposed in Figure 1 and explaining the playable elements introduced according to the taxonomy of Bedwell et al. (2012). This will make it easier to group the conclusions obtained and to perform meta-analyses to identify what is and what is not suitable in the design of GRA for personnel selection.

We will now delve into the different areas identified during the systematic review. Regarding theoretical issues, we believe that further development of GRA is necessary, at least in two ways: (1) the literature uses different terms that may overlap (e.g., serious games, gamified assessment) that need clarification. The present article may help, but the literature development should be accompanied by new terms; and (2) gamification science should develop its application to organizational psychology, proposing models linking game purposes (e.g., personnel selection, onboarding, training) with elements that make up the gaming experience to direct game design.

Concerning validity, researchers must investigate how to improve the construct validity of GRA, as well as perform more studies on predictive and discriminant validity. With regard to construct validity, we agree with Wu et al.’s (2022) recommendation to pay attention to the design of the game. In this sense, the decision to introduce some elements of Bedwell et al.’s (2012) taxonomy seems to have a differential effect on construct validity (e.g., Landers et al., 2020; Harman and Brown, 2022; Landers and Collmus, 2022). Research can deepen this line, verifying elements’ positive or negative impact on this type of validity. In the case of predictive validity, it is necessary to increase the number of studies with workers and criteria other than academic performance. In the case of job performance, dimensions other than task and contextual performance can also be analyzed, such as counterproductive work behaviors, adaptive performance, or safety performance (Ramos-Villagrasa et al., 2019).

In relation to the research on applicant reactions, although it has been the most fruitful line, there are still many unresolved issues. First, it is necessary to continue delving into the determinants of these reactions. The effect of game-framing (Collmus and Landers, 2019; McChesney et al., 2022) and the modest effect sizes found by Landers et al. (2021) caution us to be skeptical of the improvement of applicant reactions compared with those proposed by traditional tests. However, studies like those of Georgiou (2021) and Georgiou and Lievens (2022) suggest that we should continue investigating the underlying mechanisms of the GRA-reactions relationship and how to improve it. As for personal determinants, we must continue to identify the variables that determine more favorable reactions, such as being male, having experience playing video games, or self-efficacy for technology (Buil et al., 2020; Ellison et al., 2020; Gkorezis et al., 2021). This could help determine the selection processes in which it may be especially advisable to resort to GRA. For example, in the ICT sector, characterized by a majority of male professionals, all competent in technology, the use of GRA can cause the company to be considered more attractive and thus, capture talent (Aguado et al., 2019). Nor should the influence of cultural factors be forgotten (Balcerak and Woźniak, 2021), and it is advisable to conduct research in different contexts and cultures, as many marketed GRA are already offered in various languages.

The design of GRA is possibly the avenue that can offer the most development opportunities, benefiting from interdisciplinary research. Input from experts in game design can help create serious games by gameful design assessment, and data scientists can help collect and analyze the data generated by GRA in novel ways. These results will lead to new research in the other areas (validity, personal characteristics, etc.), which will enrich our knowledge about GRA and personnel selection.

Research on personal characteristics is far from conclusive. The natural advancement of GRA research, accompanied by greater terminological clarity (e.g., type of GRA, predictors it evaluates, etc.), will help clarify the influence of variables such as sex, age, or experience with computers and video games. At present, we recommend caution to practitioners in using GRA in their selection processes. Research on the adverse impact and faking follows the same line, and more investigations are necessary to determine a possible general pattern in GRA, or possible differences according to the type of GRA or the constructs evaluated.

Lastly, prevention of faking is also an issue that deserves further research. Georgiou (2021) has shown that prior explanations can influence applicants’ perception of faking, but we still need to know: (1) whether GRA use really prevents faking; (2) under what circumstances it does so or how to enhance this effect (e.g., with or without prior explanations).

### Conclusion

In recent years GRA has been presented as the “philosopher’s stone” of selection methods. The results obtained by research so far are not so optimistic, but they do prove that GRA have the potential to become one more method among those used in personnel selection. This requires an effort from both theoretical and empirical research. Fortunately, this review also shows that there are competent researchers capable of undertaking this effort.

## Data availability statement

The original contributions presented in the study are included in the article/Supplementary material, further inquiries can be directed to the corresponding author.

## Author contributions

PR-V and EF-d-R contributed to the conception and design of the study. PR-V performed the search for the systematic review. PR-V, EF-d-R, and ÁC wrote the draft of the manuscript. All authors contributed to manuscript revision, read and approved the submitted version.

## Funding

This work was supported by the Ministry of Science and Innovation, Government of Spain, under grant PID2021-122867NA-I00; and the Government of Aragon (Group S31_20D), Department of Innovation, Research and University and FEDER 2014–2020, Building Europe from Aragón.

## Conflict of interest

The authors declare that the research was conducted in the absence of any commercial or financial relationships that could be construed as a potential conflict of interest.

## Publisher’s note

All claims expressed in this article are solely those of the authors and do not necessarily represent those of their affiliated organizations, or those of the publisher, the editors and the reviewers. Any product that may be evaluated in this article, or claim that may be made by its manufacturer, is not guaranteed or endorsed by the publisher.

## References

[ref1] Aguado D. Andrés J. C. García-Izquierdo A. L. Rodríguez J. (2019). LinkedIn “big four”: job performance validation in the ICT sector. J. Work Organ. Psychol. 35, 53–64. doi: 10.5093/jwop2019a7

[ref2] Albadán J. Gaona P. Montenegro C. González-Crespo R. Herrera-Viedma E. (2018). Fuzzy logic models for non-programmed decision-making in personnel selection processes based on gamification. Informatica 29, 1–20. doi: 10.15388/Informatica.2018.155

[ref3] Albadán J. Garcia Gaona P. A. Montenegro Marin C. (2016). Assessment model in a selection process based in gamification. IEEE Lat. Am. Trans. 14, 2789–2794. doi: 10.1109/TLA.2016.7555256

[ref4] al-Qallawi S. Raghavan M. (2022). A review of online reactions to game-based assessment mobile applications. Int. J. Sel. Assess. 30, 14–26. doi: 10.1111/ijsa.12346

[ref5] Armstrong M. Ferrell J. Collmus A. Landers R. (2016). Correcting misconceptions about gamification of assessment: more than SJTs and badges. Ind. Organ. Psychol. 9, 671–677. doi: 10.1017/iop.2016.69

[ref6] Armstrong M. B. Landers R. N. (2017). An evaluation of gamified training: using narrative to improve reactions and learning. Simul. Gaming 48, 513–538. doi: 10.1177/1046878117703749

[ref7] Armstrong M. B. Landers R. N. (2018). Gamification of employee training and development: gamification of employee training. Int. J. Train. Dev. 22, 162–169. doi: 10.1111/ijtd.12124

[ref8] Auer E. M. Mersy G. Marin S. Blaik J. Landers R. N. (2022). Using machine learning to model trace behavioral data from a game-based assessment. Int. J. Sel. Assess. 30, 82–102. doi: 10.1111/ijsa.12363

[ref9] Balcerak A. Woźniak J. (2021). Reactions to some ICT-based personnel selection tools. Econ. Soc. 14, 214–231. doi: 10.14254/2071-789X.2021/14-1/14

[ref10] Bedwell W. L. Pavlas D. Heyne K. Lazzara E. H. Salas E. (2012). Toward a taxonomy linking game attributes to learning: an empirical study. Simul. Gaming 43, 729–760. doi: 10.1177/1046878112439444

[ref11] Brown M. I. Speer A. B. Tenbrink A. P. Chabris C. F. (2022). Using game-like animations of geometric shapes to simulate social interactions: an evaluation of group score differences. Int. J. Sel. Assess. 30, 167–181. doi: 10.1111/ijsa.12375, PMID: 35935096 PMC9355331

[ref12] Buil I. Catalán S. Martínez E. (2020). Understanding applicants’ reactions to gamified recruitment. J. Bus. Res. 110, 41–50. doi: 10.1016/j.jbusres.2019.12.041

[ref13] Chamorro-Premuzic T. Akhtar R. Winsborough D. Sherman R. A. (2017). The datafication of talent: how technology is advancing the science of human potential at work. Curr. Opin. Behav. Sci. 18, 13–16. doi: 10.1016/j.cobeha.2017.04.007

[ref14] Collmus A. B. Landers R. N. (2019). Game-framing to improve applicant perceptions of cognitive assessments. J. Pers. Psychol. 18, 157–162. doi: 10.1027/1866-5888/a000227

[ref15] Connelly L. Burbach B. E. Kennedy C. Walters L. (2018). Escape room recruitment event: description and lessons learned. J. Nurs. Educ. 57, 184–187. doi: 10.3928/01484834-20180221-12, PMID: 29505080

[ref16] Coovert M. D. Wiernik B. M. Martin J. (2020). Use of technology enhanced simulations for cyber aptitude assessment: Phase II prototype development. MCD and Associates Plant City United States. Available at: https://apps.dtic.mil/sti/citations/AD1107016

[ref17] Egol K. A. Schwarzkopf R. Funge J. Gray J. Chabris C. Jerde T. E. . (2017). Can video game dynamics identify orthopaedic surgery residents who will succeed in training? Int. J. Med. Educ. 8, 123–125. doi: 10.5116/ijme.58e3.c236, PMID: 28412723 PMC5440060

[ref18] Ellison L. J. McClure Johnson T. Tomczak D. Siemsen A. Gonzalez M. F. (2020). Game on! Exploring reactions to game-based selection assessments. J. Manag. Psychol. 35, 241–254. doi: 10.1108/JMP-09-2018-0414

[ref19] Fetzer M. McNamara J. Geimer J. L. (2017). “Gamification, serious games and personnel selection,” in The Wiley Blackwell Handbook of the Psychology of Recruitment, Selection and Employee Retention. 1st Edn. eds. Goldstein H. W. Pulakos E. D. Passmore J. Semedo C. (Hoboken, NY: Wiley), 293–309.

[ref20] Formica E. Gaiffi E. Magnani M. Mancini A. Scatolini E. Ulivieri M. (2017). Can video games be an innovative tool to assess personality traits of the millennial generation? An exploratory research. BPA 280, 29–47.

[ref21] García-Izquierdo A. L. Ramos-Villagrasa P. J. Lubiano M. A. (2020). Developing biodata for public manager selection purposes: a comparison between fuzzy logic and traditional methods. J. Work Organ. Psychol. 36, 231–242. doi: 10.5093/jwop2020a22

[ref22] Georgiou K. (2021). Can explanations improve applicant reactions towards gamified assessment methods? Int. J. Sel. Assess. 29, 253–268. doi: 10.1111/ijsa.12329

[ref23] Georgiou K. Gouras A. Nikolaou I. (2019). Gamification in employee selection: the development of a gamified assessment. Int. J. Sel. Assess. 27, 91–103. doi: 10.1111/ijsa.12240

[ref24] Georgiou K. Lievens F. (2022). Gamifying an assessment method: what signals are organizations sending to applicants? J. Manager. Psychol 37, 559–574. doi: 10.1108/JMP-12-2020-0653

[ref25] Georgiou K. Nikolaou I. (2020). Are applicants in favor of traditional or gamified assessment methods? Exploring applicant reactions towards a gamified selection method. Comput. Hum. Behav. 109:106356. doi: 10.1016/j.chb.2020.106356

[ref26] Gkorezis P. Georgiou K. Nikolaou I. Kyriazati A. (2021). Gamified or traditional situational judgement test? A moderated mediation model of recommendation intentions via organizational attractiveness. Eur. J. Work Organ. Psy. 30, 240–250. doi: 10.1080/1359432X.2020.1746827

[ref27] Harman J. L. Brown K. D. (2022). Illustrating a narrative: a test of game elements in game-like personality assessment. Int. J. Sel. Assess. 30, 157–166. doi: 10.1111/ijsa.12374

[ref28] Hilliard A. Kazim E. Bitsakis T. Leutner F. (2022). Measuring personality through images: validating a forced-choice image-based assessment of the big five personality traits. J. Intelligence 10:12. doi: 10.3390/jintelligence10010012, PMID: 35225927 PMC8883940

[ref29] Hommel B. E. Ruppel R. Zacher H. (2022). Assessment of cognitive flexibility in personnel selection: validity and acceptance of a gamified version of the Wisconsin Card Sorting Test. Int. J. Sel. Assess. 30, 126–144. doi: 10.1111/ijsa.12362

[ref30] Korn O. Brenner F. Börsig J. Lalli F. Mattmüller M. Müller A. (2018). “Defining recrutainment: a model and a survey on the gamification of recruiting and human resources,” in Advances in the Human Side of Service Engineering. Vol. 601. eds. Freund L. E. Cellary W. (Cham: Springer International Publishing), 37–49.

[ref01] Küpper D. M. Klein K. Völckner F. (2021). Gamifying employer branding: An integrating framework and research propositions for a new HRM approach in the digitized economy. Hum. Resour. Manag. Rev. 31:100686. doi: 10.1016/j.hrmr.2019.04.002

[ref31] Landers R. N. Armstrong M. B. Collmus A. B. Mujcic S. Blaik J. (2021). Theory-driven game-based assessment of general cognitive ability: design theory, measurement, prediction of performance, and test fairness. J. Appl. Psychol. doi: 10.1037/apl0000954 [Epub ahead of print]., PMID: 34672652

[ref32] Landers R. N. Auer E. M. Abraham J. D. (2020). Gamifying a situational judgment test with immersion and control game elements: effects on applicant reactions and construct validity. J. Manag. Psychol. 35, 225–239. doi: 10.1108/JMP-10-2018-0446

[ref33] Landers R. N. Auer E. M. Collmus A. B. Armstrong M. B. (2018). Gamification science, its history and future: definitions and a research agenda. Simul. Gaming 49, 315–337. doi: 10.1177/1046878118774385

[ref34] Landers R. N. Collmus A. B. (2022). Gamifying a personality measure by converting it into a story: convergence, incremental prediction, faking, and reactions. Int. J. Sel. Assess. 30, 145–156. doi: 10.1111/ijsa.12373

[ref35] Landers R. N. Sanchez D. R. (2022). Game-based, gamified, and gamefully designed assessments for employee selection: definitions, distinctions, design, and validation. Int. J. Sel. Assess. 30, 1–13. doi: 10.1111/ijsa.12376

[ref36] Laumer S. Eckhardt A. Weitzel T. (2012). Online gaming to find a new job–examining job seekers’ intention to use serious games as a self-assessment tool. German J. Hum. Res. Manag. 26, 218–240. doi: 10.1177/239700221202600302

[ref37] McChesney J. Campbell C. Wang J. Foster L. (2022). What is in a name? Effects of game-framing on perceptions of hiring organizations. Int. J. Sel. Assess. 30, 182–192. doi: 10.1111/ijsa.12370

[ref38] McCord J.-L. Harman J. L. Purl J. (2019). Game-like personality testing: an emerging mode of personality assessment. Personal. Individ. Differ. 143, 95–102. doi: 10.1016/j.paid.2019.02.017

[ref39] Melchers K. G. Basch J. M. (2022). Fair play? Sex-, age-, and job-related correlates of performance in a computer-based simulation game. Int. J. Sel. Assess. 30, 48–61. doi: 10.1111/ijsa.12337

[ref40] Nacke L. Deterding S. (2017). The maturing of gamification research. Comput. Hum. Behav. 71, 450–454. doi: 10.1016/j.chb.2016.11.062

[ref41] Nikolaou I. (2021). What is the role of Technology in Recruitment and Selection? Span. J. Psychol. 24:e2. doi: 10.1017/SJP.2021.6, PMID: 33536110

[ref42] Nikolaou I. Georgiou K. Kotsasarlidou V. (2019). Exploring the relationship of a gamified assessment with performance. Span. J. Psychol. 22:E6. doi: 10.1017/sjp.2019.5, PMID: 30819261

[ref43] Page M. J. McKenzie J. E. Bossuyt P. M. Boutron I. Hoffmann T. C. Mulrow C. D. . (2021). The PRISMA 2020 statement: an updated guideline for reporting systematic reviews. BMJ 372, 1–9. doi: 10.1136/bmj.n71, PMID: 33782057 PMC8005924

[ref44] Peters H. Kyngdon A. Stillwell D. (2021). Construction and validation of a game-based intelligence assessment in minecraft. Comput. Hum. Behav. 119:106701. doi: 10.1016/j.chb.2021.106701

[ref45] Quiroga M. A. Diaz A. Román F. J. Privado J. Colom R. (2019). Intelligence and video games: beyond “brain-games”. Intelligence 75, 85–94. doi: 10.1016/j.intell.2019.05.001

[ref46] Ramos-Villagrasa P. J. Barrada J. R. Fernández-del-Río E. Koopmans L. (2019). Assessing job performance using brief self-report scales: the case of the individual work performance questionnaire. J. Work Organ. Psychol. 35, 195–205. doi: 10.5093/jwop2019a21

[ref47] Salgado J. F. Moscoso S. García-Izquierdo A. L. Anderson N. R. (2017). “Inclusive and discrimination-free personnel selection,” in Shaping Inclusive Workplaces Through Social Dialogue. eds. Arenas A. Di D. Marco Munduate L. Euwema M. C. (Cham: Springer International Publishing), 103–119.

[ref48] Sanchez D. R. Weiner E. Van Zelderen A. (2022). Virtual reality assessments (VRAs): exploring the reliability and validity of evaluations in VR. Int. J. Sel. Assess. 30, 103–125. doi: 10.1111/ijsa.12369

[ref49] Schalken N. Rietbergen C. (2017). The reporting quality of systematic reviews and meta-analyses in industrial and organizational psychology: a systematic review. Front. Psychol. 8:1395. doi: 10.3389/fpsyg.2017.01395, PMID: 28878704 PMC5572251

[ref50] Tippins N. T. (2015). Technology and assessment in selection. Annu. Rev. Organ. Psych. Organ. Behav. 2, 551–582. doi: 10.1146/annurev-orgpsych-031413-091317

[ref51] Wiernik B. M. Raghavan M. Caretta T. R. Coovert M. D. (2022). Developing and validating a serious game-based assessment for cyber occupations in the US Air Force. Int. J. Sel. Assess. 30, 27–47. doi: 10.1111/ijsa.12378

[ref52] Woods S. A. Ahmed S. Nikolaou I. Costa A. C. Anderson N. R. (2020). Personnel selection in the digital age: a review of validity and applicant reactions, and future research challenges. Eur. J. Work Organ. Psy. 29, 64–77. doi: 10.1080/1359432X.2019.1681401

[ref53] Wu F. Y. Mulfinger E. Alexander L. Sinclair A. L. McCloy R. A. Oswald F. L. (2022). Individual differences at play: an investigation into measuring big five personality facets with game-based assessments. Int. J. Sel. Assess. 30, 62–81. doi: 10.1111/ijsa.12360

