# Supplementary material for: Game-related assessments for personnel selection: A systematic review
Source: Front Psychol. 2022 Sep 28;13:952002. doi: 10.3389/fpsyg.2022.952002 (PMC9554090; doi:10.3389/fpsyg.2022.952002)
Supplement: Supplementary file 1 [file Presentation_1.pdf]

Supplementary Material – 1

Full list of articles analyzed in screening

Albadán, J., Gaona, P., Montenegro, C., González-Crespo, R., & Herrera-Viedma, E. (2018). Fuzzy Logic Models for Non-Programmed Decision-Making in Personnel Selection Processes Based on Gamification. *Informatica*, *29*(1), 1–20. https://doi.org/10.15388/Informatica.2018.155

Balcerak, A., & Woźniak, J. (2021). Reactions to some ICT-based personnel selection tools. *Economics & Sociology*, *14*(1), 214–231. https://doi.org/10.14254/2071-789X.2021/14-1/14

Baležentis, T., & Zeng, S. (2013). Group multi-criteria decision making based upon interval-valued fuzzy numbers: An extension of the MULTIMOORA method. *Expert Systems with Applications*, *40*(2), 543–550. https://doi.org/10.1016/j.eswa.2012.07.066

Bangerter, A., Roulin, N., & König, C. J. (2012). Personnel selection as a signaling game. *Journal of Applied Psychology*, *97*(4), 719–738. https://doi.org/10.1037/a0026078

Bello, M., Bello, R., García-Lorenzo, M. M., & Nowe, A. (2016). Personnel Selection in a Competitive Environment. *Computación y Sistemas*, *20*(2). https://doi.org/10.13053/cys-20-2-2315

Berriochoa, C., Ward, M. C., Weller, M. A., Holliday, E., Kusano, A., Thomas, C. R., & Tendulkar, R. D. (2016). Applicant Interview Experiences and Postinterview Communication of the 2016 Radiation Oncology Match Cycle. *International Journal of Radiation Oncology*Biology*Physics*, *96*(3), 514–520. https://doi.org/10.1016/j.ijrobp.2016.08.009

Bertuglia, L. E. (1994). Exploration of the glass ceiling. *SWE Magazine, 40*, 27-29.

Blasier, R. D., Gagnon, M. R., Iannotti, J. P., & Jarvis-Selinger, S. (2014). Hiring Your Next Partner: AOA Critical Issues. *Journal of Bone and Joint Surgery*, *96*(17), e150. https://doi.org/10.2106/JBJS.M.00725

Bogdanovic, D., & Miletic, S. (2014). Personnel evaluation and selection by multicriteria decision making method. *Economic Computation & Economic Cybernetics Studies & Research, 48*, 22-39.

Book Reviews. (1964). *Personnel Psychology*, *17*(4), 449–478. https://doi.org/10.1111/j.1744-6570.1964.tb00080.x

Bozionelos, N. (2005). When the inferior candidate is offered the job: The selection interview as a political and power game. *Human Relations*, *58*(12), 1605–1631. https://doi.org/10.1177/0018726705061437

Buchan, J. (1992). The numbers game. *Nursing Times*, *88*(39), 19.

Buchan, J. (2000). Rethink the weighting game. *Nursing Standard (Royal College of Nursing (Great Britain): 1987)*, *14*(46), 23. https://doi.org/10.7748/ns.14.46.23.s38

Burak, E., & Mustafa, K. (2018). A systematic approach for an application of personnel selection in assembly line balancing problem. *International Transactions in Operational Research, 25*, 1001-1025. https://doi.org/10.1111/itor.12439

Cain, J., Scott, D. R., & Smith, K. (2010). Use of social media by residency program directors for resident selection. *American Journal of Health-System Pharmacy*, *67*(19), 1635–1639. https://doi.org/10.2146/ajhp090658

Caisucar, M., Naik Dessai, A., & Usgaonkar, G. (2021). Validation of portfolio allocation in NPD: Fuzzy-TOPSIS and COPRAS-grey approach. *International Journal of System Assurance Engineering and Management*, *12*(1), 37–43. https://doi.org/10.1007/s13198-020-01024-4

Çelikbilek, Y. (2018). A grey analytic hierarchy process approach to project manager selection. *Journal of Organizational Change Management*, *31*(3), 749–765. https://doi.org/10.1108/JOCM-04-2017-0102

Chai, J., Ngai, E. W. T., & Liu, J. N. K. (2014). Dynamic tolerant skyline operation for decision making. *Expert Systems with Applications*, *41*(15), 6890–6903. https://doi.org/10.1016/j.eswa.2014.04.041

Chang, K.-L. (2015). The Use of a Hybrid MCDM Model for Public Relations Personnel Selection. *Informatica*, *26*(3), 389–406. https://doi.org/10.15388/Informatica.2015.54

Chen, C-T., Pai, P-F., & Hung, W-Z. (2013). A New Decision-Making Process for Selecting Project Leader Based on Social Network and Knowledge Map. *International Journal of Fuzzy Systems, 15*, 36-46.

Clancy, S. (2006). You gotta know when to hold ’em.. *Dental Assistant (Chicago, Ill.: 1994)*, *75*(3), 30–33.

Clare, A. S., Cummings, M. L., & Repenning, N. P. (2015). Influencing Trust for Human–Automation Collaborative Scheduling of Multiple Unmanned Vehicles. *Human Factors: The Journal of the Human Factors and Ergonomics Society*, *57*(7), 1208–1218. https://doi.org/10.1177/0018720815587803

Collmus, A. B., & Landers, R. N. (2019). Game-Framing to Improve Applicant Perceptions of Cognitive Assessments. *Journal of Personnel Psychology*, *18*(3), 157–162. https://doi.org/10.1027/1866-5888/a000227

Connelly, L., Burbach, B. E., Kennedy, C., & Walters, L. (2018). Escape Room Recruitment Event: Description and Lessons Learned. *Journal of Nursing Education*, *57*(3), 184–187. https://doi.org/10.3928/01484834-20180221-12

Cummings, M. L., Clare, A., & Hart, C. (2010). The Role of Human-Automation Consensus in Multiple Unmanned Vehicle Scheduling. *Human Factors: The Journal of the Human Factors and Ergonomics Society*, *52*(1), 17–27. https://doi.org/10.1177/0018720810368674

Deliktas, D., & Ustun, O. (2017). Student selection and assignment methodology based on fuzzy MULTIMOORA and multichoice goal programming. *International Transactions in Operational Research*, *24*(5), 1173–1195. https://doi.org/10.1111/itor.12185

Do you know how to play the hiring game. (2000). *Hospital Peer Review*, *25*(11), suppl 3-4.

Dodangeh, J., Sorooshian, S., & Afshari, A. R. (2014). Linguistic Extension for Group Multicriteria Project Manager Selection. *Journal of Applied Mathematics*, *2014*, 1–8. https://doi.org/10.1155/2014/570398

Dodd, L., & Smith, J. Q. (2013). Devolving command decisions in complex operations. *Journal of the Operational Research Society*, *64*(1), 17–33. https://doi.org/10.1057/jors.2012.7

Egol, K. A., Schwarzkopf, R., Funge, J., Gray, J., Chabris, C., Jerde, T. E., & Strauss, E. J. (2017). Can video game dynamics identify orthopaedic surgery residents who will succeed in training? *International Journal of Medical Education*, *8*, 123–125. https://doi.org/10.5116/ijme.58e3.c236

Ellison, L. J., McClure Johnson, T., Tomczak, D., Siemsen, A., & Gonzalez, M. F. (2020). Game on! Exploring reactions to game-based selection assessments. *Journal of Managerial Psychology*, *35*(4), 241–254. https://doi.org/10.1108/JMP-09-2018-0414

Eltoukhy, A. E. E., Wang, Z. X., Chan, F. T. S., & Chung, S. H. (2018). Joint optimization using a leader–follower Stackelberg game for coordinated configuration of stochastic operational aircraft maintenance routing and maintenance staffing. *Computers & Industrial Engineering*, *125*, 46–68. https://doi.org/10.1016/j.cie.2018.08.012

Forbes, J., & Brichko, L. (2017). Selection into Emergency Medicine fellowship training: Is it a fair game?: TRAINEE FOCUS. *Emergency Medicine Australasia*, *29*(4), 456–458. https://doi.org/10.1111/1742-6723.12825

Formica, E., Gaiffi, E., Magnani, M., Mancini, A., Scatolini, E., & Ulivieri, M. (2017). Can video games be an innovative tool to assess personality traits of the Millennial generation? An exploratory research. *Bollettino di Psicologia Applicata*, *280*, 29-47.

Garg, H., & Kaur, G. (2020). A robust correlation coefficient for probabilistic dual hesitant fuzzy sets and its applications. *Neural Computing and Applications, 32*, 8847-8866. https://doi.org/10.1007/s00521-019-04362-y

Georgiou, K., & Nikolaou, I. (2020). Are applicants in favor of traditional or gamified assessment methods? Exploring applicant reactions towards a gamified selection method. *Computers in Human Behavior*, *109*, 106356. https://doi.org/10.1016/j.chb.2020.106356

Gil, S. M., Gil, J., Ruiz, F., Irazusta, A., & Irazusta, J. (2007). Physiological and Anthropometric Characteristics of Young Soccer Players According to Their Playing Position: Relevance for the Selection Process. *The Journal of Strength and Conditioning Research*, *21*(2), 438. https://doi.org/10.1519/R-19995.1

Green, B. S., Blake, C., & Caulfield, B. M. (2011). A Valid Field Test Protocol of Linear Speed and Agility in Rugby Union. *Journal of Strength and Conditioning Research*, *25*(5), 1256–1262. https://doi.org/10.1519/JSC.0b013e3181d8598b

Greene, J. (1999). Head games. *Materials management in health care, 8*, 42.

Gürbüz, T., & Albayrak, Y. E. (2014). An engineering approach to human resources performance evaluation: Hybrid MCDM application with interactions. *Applied Soft Computing*, *21*, 365–375. https://doi.org/10.1016/j.asoc.2014.03.025

Hilliard, A., Kazim, E., Bitsakis, T., & Leutner, F. (2022). Measuring Personality through Images: Validating a Forced-Choice Image-Based Assessment of the Big Five Personality Traits. *Journal of Intelligence*, *10*(1), 12. https://doi.org/10.3390/jintelligence10010012

Holdren, R. C. (1992). Making practice retirement acquisition work for you. *Medical Group Management Journal*, *39*(1), 56–58, 60, 62–63.

Hommel, B. E., Ruppel, R., & Zacher, H. (2022). Assessment of cognitive flexibility in personnel selection: Validity and acceptance of a gamified version of the Wisconsin Card Sorting Test. *International Journal of Selection and Assessment*, *30*(1), 126–144. https://doi.org/10.1111/ijsa.12362

Jennings, J., Wundersitz, D. W., Sullivan, C. J., Cousins, S. D., Tehan, G., & Kingsley, M. I. (2021). Physical testing characteristics better explain draft outcome than in-game movement profile in junior elite Australian rules football players. *Journal of Science and Medicine in Sport*, *24*(12), 1284–1289. https://doi.org/10.1016/j.jsams.2021.07.005

Ji, P., Zhang, Hy. & Wang, Jq. (2018). A projection-based TODIM method under multi-valued neutrosophic environments and its application in personnel selection. *Neural Computing and Applications, 29*, 221–234. https://doi.org/10.1007/s00521-016-2436-z

Jossi, F. (2005). Big game hunting. In a CIO search, look inside and outside the company for business and people skills. *Healthcare Informatics: The Business Magazine for Information and Communication Systems*, *22*(10), 39–40, 42.

Kevern, J., & Webb, C. (2004). Mature women's experiences of preregistration nurse education. *Journal of Advanced Nursing*, *45*(3), 297–306. https://doi.org/10.1046/j.1365-2648.2003.02890.x

Khan, A. A., Shameem, M., Kumar, R. R., Hussain, S., & Yan, X. (2019). Fuzzy AHP based prioritization and taxonomy of software process improvement success factors in global software development. *Applied Soft Computing*, *83*, 105648. https://doi.org/10.1016/j.asoc.2019.105648

Kleiboer, M. (1997). Simulation Methodology for Crisis Management Support. *Journal of Contingencies and Crisis Management*, *5*(4), 198–206. https://doi.org/10.1111/1468-5973.00057

Konacoglu, J., & Albaryrak, I. (2019). A new fuzzy decision making approach for personnel selection problem. *Intelligent Decision Technologies, 12*, 471-482. https://doi.org/10.3233/IDT-180350

Kubera, B., Klement, J., Wagner, C., Rädel, C., Eggeling, J., Füllbrunn, S., Kaczmarek, M. C., Levinsky, R., & Peters, A. (2016). Differences in fairness and trust between lean and corpulent men. *International Journal of Obesity*, *40*(11), 1802–1808. https://doi.org/10.1038/ijo.2016.134

Kuzmits, F. E., & Adams, A. J. (2008). The NFL Combine: Does It Predict Performance in the National Football League? *Journal of Strength and Conditioning Research*, *22*(6), 1721–1727. https://doi.org/10.1519/JSC.0b013e318185f09d

LaViolette, S. (1979). Hospitals change registry game plan. *Modern Healthcare*, *9*(5), 38–39.

Lee, R. (2002). The guide to good staff. *Quality World, 28*, 36-38.

Li, D.-F. (2007). A fuzzy closeness approach to fuzzy multi-attribute decision making. *Fuzzy Optimization and Decision Making*, *6*(3), 237–254. https://doi.org/10.1007/s10700-007-9010-1

Liang, G.-S., & Wang, M.-J. J. (1994). Personnel selection using fuzzy MCDM algorithm. *European Journal of Operational Research*, *78*(1), 22–33. https://doi.org/10.1016/0377-2217(94)90119-8

Lievens, F., & de Soete, B. (2011). Instruments for personnel selection in the 21st Century: Research and practice [Instrumenten om Personeel te Selecteren in de 21ste Eeuw: Onderzoek en Praktijk]. *Gedrag en Organisatie, 24*, 18-42.

Luo, S., & Xing, L. (2019). A Hybrid Decision Making Framework for Personnel Selection Using BWM, MABAC and PROMETHEE. *International Journal of Fuzzy Systems*, *21*(8), 2421–2434. https://doi.org/10.1007/s40815-019-00745-4

Mandelbaum, M., & Taub, P. J. (2020). "The Name Game": A Case against Hidden Identity on Social Media during the Plastic Surgery Interview Season. *Plastic & Reconstructive Surgery*, *146*(2), 245e–246e. https://doi.org/10.1097/PRS.0000000000007030

Martin, P. (2005). The numbers game. NHS Scotland's chief nurse says the country is on track to recruit 12,000 new nurses. *Nursing Standard (Royal College of Nursing (Great Britain): 1987)*, *19*(48), 16. https://doi.org/10.7748/ns.19.48.16.s19

Martínez-Pernía, D., Núñez-Huasaf, J., del Blanco, Á., Ruiz-Tagle, A., Velásquez, J., Gomez, M., Robert Blesius, C., Ibañez, A., Fernández-Manjón, B., & Slachevsky, A. (2017). Using game authoring platforms to develop screen-based simulated functional assessments in persons with executive dysfunction following traumatic brain injury. *Journal of Biomedical Informatics*, *74*, 71–84. https://doi.org/10.1016/j.jbi.2017.08.012

McGovern, P. (2000). The Irish brawn drain: English League clubs and Irish footballers, 1946-1995. *British Journal of Sociology*, *51*(3), 401–418. https://doi.org/10.1080/00071310050131594

McKinley, R. A., McIntire, L. K., & Funke, M. A. (2011). Operator Selection for Unmanned Aerial Systems: Comparing Video Game Players and Pilots. *Aviation, Space, and Environmental Medicine*, *82*(6), 635–642. https://doi.org/10.3357/ASEM.2958.2011

Melchers, K. G., & Basch, J. M. (2022). Fair play? Sex-, age-, and job-related correlates of performance in a computer-based simulation game. *International Journal of Selection and Assessment*, *30*(1), 48–61. https://doi.org/10.1111/ijsa.12337

Meyer, C. T. (1994). The osteopathic medicine game: New strategies for winning. *The Journal of the American Osteopathic Association*, *94*(9), 715–718, 723–731.

Miyata, M., Sung, S.-C., & Aoyama Gakuin University, 5-10-1 Fuchinobe, Chuo-ku, Kanagawa 252-5258, Japan. (2012). Coalition Formation Based Staffing Strategy Development. *Journal of Advanced Computational Intelligence and Intelligent Informatics*, *16*(3), 430–435. https://doi.org/10.20965/jaciii.2012.p0430

Moore, A. (2004). Cancer care. Diagnosis. The waiting game. *The Health Service Journal, 114* (5933), 4-6.

Nabeeh, N. A., Smarandache, F., Abdel-Basset, M., El-Ghareeb, H. A., & Aboelfetouh, A. (2019). An Integrated Neutrosophic-TOPSIS Approach and Its Application to Personnel Selection: A New Trend in Brain Processing and Analysis. *IEEE Access*, *7*, 29734–29744. https://doi.org/10.1109/ACCESS.2019.2899841

Neal, J. M. (2016). SUCCESSFUL INTERVIEWING: DON'T LOSE THE BIG GAME. *Physician Leadership Journal*, *3*(1), 18–21.

Nikolaou, I. (2021). What is the Role of Technology in Recruitment and Selection? *The Spanish Journal of Psychology*, *24*, e2. https://doi.org/10.1017/SJP.2021.6

Nikolaou, I., Georgiou, K., & Kotsasarlidou, V. (2019). Exploring the Relationship of a Gamified Assessment with Performance. *The Spanish Journal of Psychology*, *22*, E6. https://doi.org/10.1017/sjp.2019.5

O'Connor, S. (1999). Reference checking: Don't get lost in the translation. *Michigan Health & Hospitals*, *35*(6), 18.

Otto, J. M. (2012). Changing the game: What CFOs should know about physician compensation. *Healthcare Financial Management: Journal of the Healthcare Financial Management Association*, *66*(10), 70–74, 76.

Petridis, K., Drogalas, G., & Zografidou, E. (2021). Internal auditor selection using a TOPSIS/non-linear programming model. *Annals of Operations Research*, *296*(1–2), 513–539. https://doi.org/10.1007/s10479-019-03307-x

Pitchai, A., Reddy A. V., & Savarimuthu, N. (2016). Fuzzy based Quantum Genetic Algorithm for Project Team Formation: *International Journal of Intelligent Information Technologies*, *12*(1), 31–46. https://doi.org/10.4018/IJIIT.2016010102

Qin, J., Liu, X., & Pedrycz, W. (2016). Frank aggregation operators and their application to hesitant fuzzy multiple attribute decision making. *Applied Soft Computing*, *41*, 428–452. https://doi.org/10.1016/j.asoc.2015.12.030

Randolph, L. B. (2003). The Numbers Game: *Nursing Management (Springhouse)*, *34*, 7–8. https://doi.org/10.1097/00006247-200307004-00003

Rani, P., Mishra, A. R., Krishankumar, R., Mardani, A., Cavallaro, F., Soundarapandian Ravichandran, K., & Balasubramanian, K. (2020). Hesitant Fuzzy SWARA-Complex

Proportional Assessment Approach for Sustainable Supplier Selection (HF-SWARA-COPRAS). *Symmetry*, *12*(7), 1152. https://doi.org/10.3390/sym12071152

Rossignol, P. L., Gabbett, T. J., Comerford, D., & Stanton, W. R. (2014). Repeated-Sprint Ability and Team Selection in Australian Football League Players. *International Journal of Sports Physiology and Performance*, *9*(1), 161–165. https://doi.org/10.1123/ijspp.2013-0005

Russell, J., & Havel, S. (2010). Candidate marketing takes the guessing game out of choosing employers. *Nursing Economic$*, *28*(3), 195–197.

Samanlioglu, F., Taskaya, Y. E., Gulen, U. C., & Cokcan, O. (2018). A Fuzzy AHP–TOPSIS-Based Group Decision-Making Approach to IT Personnel Selection. *International Journal of Fuzzy Systems*, *20*(5), 1576–1591. https://doi.org/10.1007/s40815-018-0474-7

Sang, X., Liu, X., & Qin, J. (2015). An analytical solution to fuzzy TOPSIS and its application in personnel selection for knowledge-intensive enterprise. *Applied Soft Computing*, *30*, 190–204. https://doi.org/10.1016/j.asoc.2015.01.002

Satiani, B., & Williams, T. E. (2009). Fill in the blanks. 8 steps to make your practice competitive in the physician hiring game. *MGMA Connexion*, *9*(7), 36–40.

Schell, S., de Groote, J. K., Moog, P., & Hack, A. (2020). Successor selection in family business—A signaling game. *Journal of Family Business Strategy*, *11*(3), 100286. https://doi.org/10.1016/j.jfbs.2019.04.005

Sengupta, K., Abdel-Hamid, T.K., & Van Wassenhove, L.N. (2008). The experience trap. *Harvard Business Review, 8, 94-101.*

Serel, D. A., & Erel, E. (2008). Coordination of staffing and pricing decisions in a service firm. *Applied Stochastic Models in Business and Industry*, *24*(4), 307–323. https://doi.org/10.1002/asmb.708

Smillie, L. D., Quek, B.-K., & Dalgleish, L. I. (2014). The Impact of Asymmetric Partial Feedback on Response-Bias: Partial Feedback. *Journal of Behavioral Decision Making*, *27*(2), 157–169. https://doi.org/10.1002/bdm.1793

Smith, R., Lagarde, M., Blaauw, D., Goodman, C., English, M., Mullei, K., Pagaiya, N., Tangcharoensathien, V., Erasmus, E., & Hanson, K. (2013). Appealing to altruism: An alternative strategy to address the health workforce crisis in developing countries? *Journal of Public Health*, *35*(1), 164–170. https://doi.org/10.1093/pubmed/fds066

Tarakci, H., Ozdemir, Z., & Sharafali, M. (2009). On the staffing policy and technology investment in a specialty hospital offering telemedicine. *Decision Support Systems*, *46*(2), 468–480. https://doi.org/10.1016/j.dss.2008.08.001

Vere-Jones, E. (2006). The waiting game. *Nursing Times*, *102*(12), 20–21.

Verma, R. (2021). On intuitionistic fuzzy order-α divergence and entropy measures with MABAC method for multiple attribute group decision-making. *Journal of Intelligent & Fuzzy Systems*, *40*(1), 1191–1217. https://doi.org/10.3233/JIFS-201540

Vieira Campos Proença, M. T., & Valente Dias de Oliveira, E. T. (2009). From normative to tacit knowledge: CVs analysis in personnel selection. *Employee Relations*, *31*(4), 427–447. https://doi.org/10.1108/01425450910965469

von Sydow, M., Braus, N., & Hahn, U. (2019). On the ignorance of group-level effects—The tragedy of personnel evaluation? *Journal of Experimental Psychology: Applied*, *25*(3), 491–515. https://doi.org/10.1037/xap0000173

Vousden, M. (1979). Playing the interview game. *Nursing Mirror*, *149*(14), 12.

Wan, S.-P., Wang, Q.-Y., & Dong, J.-Y. (2013). The extended VIKOR method for multi-attribute group decision making with triangular intuitionistic fuzzy numbers. *Knowledge-Based Systems*, *52*, 65–77. https://doi.org/10.1016/j.knosys.2013.06.019

Weissbart, S. J., Kim, S. J., Feinn, R. S., & Stock, J. A. (2015). Relationship Between the Number of Residency Applications and the Yearly Match Rate: Time to Start Thinking About an Application Limit? *Journal of Graduate Medical Education*, *7*(1), 81–85. https://doi.org/10.4300/JGME-D-14-00270.1

Wen, T.-C., Chang, K.-H., & Lai, H.-H. (2018). Improving personnel selection by combining the minimal variance OWA operator and IPA. *Journal of Intelligent & Fuzzy Systems*, *35*(6), 6229–6239. https://doi.org/10.3233/JIFS-171686

White, C. R. (1995). Preferences in teams and hierarchies. *Production Planning & Control*, *6*(6), 500–507. https://doi.org/10.1080/09537289508930308

White, J. G. (2000). Taking your practice to a new level. Part II. *SADJ: Journal of the South African Dental Association = Tydskrif van Die Suid-Afrikaanse Tandheelkundige Vereniging*, *55*(10), 561–564.

Wiernik, B. M., Raghavan, M., Caretta, T. R., & Coovert, M. D. (2022). Developing and validating a serious game-based assessment for cyber occupations in the US Air Force. *International Journal of Selection and Assessment*, *30*(1), 27–47. https://doi.org/10.1111/ijsa.12378

Willis, W. K., Muslin, I., & Timko, K. N. (2016). A house divided: Cooperative and competitive recruitment in vital industries. *Journal of Nursing Management*, *24*(2), 253–260. https://doi.org/10.1111/jonm.12308

Woods, C. T., Veale, J. P., Collier, N., & Robertson, S. (2017). The use of player physical and technical skill match activity profiles to predict position in the Australian Football League draft. *Journal of Sports Sciences*, *35*(4), 325–330. https://doi.org/10.1080/02640414.2016.1164334

Woods, S. A., Ahmed, S., Nikolaou, I., Costa, A. C., & Anderson, N. R. (2020). Personnel selection in the digital age: A review of validity and applicant reactions, and future research challenges. *European Journal of Work and Organizational Psychology*, *29*, 64-77. https://doi.org/10.1080/1359432X.2019.1681401

Wu, F. Y., Mulfinger, E., Alexander, L., Sinclair, A. L., McCloy, R. A., & Oswald, F. L. (2022). Individual differences at play: An investigation into measuring Big Five personality facets

with game-based assessments. *International Journal of Selection and Assessment*, *30*(1), 62–81. https://doi.org/10.1111/ijsa.12360

Wu, L.-C., Chang, K.-L., & Liao, S.-K. (2020). A Hybrid MCDM Model to Select Optimal Hosts of Variety Shows in the Social Media Era. *Symmetry*, *12*(1), 125. https://doi.org/10.3390/sym12010125

Wyer, J. C. (1988). A Stochastic, Psychometric Valuation Model for Personnel Selection Systems. *Decision Sciences, 19*, 700-707. https://doi.org/10.1111/j.1540-5915.1988.tb00295.x

Xu, Y. (2021). Construction of maintenance staffing model for managing sports venues in comprehensive sports games: A research based on the practice of the 7th military world games. *The International Journal of Electrical Engineering & Education*, 002072092110052. https://doi.org/10.1177/00207209211005265

Yalçın, N., & Yapıcı Pehlivan, N. (2019). Application of the Fuzzy CODAS Method Based on Fuzzy Envelopes for Hesitant Fuzzy Linguistic Term Sets: A Case Study on a Personnel Selection Problem. *Symmetry*, *11*(4), 493. https://doi.org/10.3390/sym11040493

Yu, D., & Fang, L. (2014). Intuitionistic multiplicative aggregation operators with their application in group decision making. *Journal of Intelligent & Fuzzy Systems*, *27*(1), 131–142. https://doi.org/10.3233/IFS-130984

Yu, W., Zhang, Z., Zhong, Q., & Sun, L. (2017). Extended TODIM for multi-criteria group decision making based on unbalanced hesitant fuzzy linguistic term sets. *Computers & Industrial Engineering*, *114*, 316–328. https://doi.org/10.1016/j.cie.2017.10.029

Yue, Z. (2013). An avoiding information loss approach to group decision making. *Applied Mathematical Modelling*, *37*(1–2), 112–126. https://doi.org/10.1016/j.apm.2012.02.008

Zaremski, J. L., Rao, A., Myers, R., Mautner, K., Berkoff, D., Ross, D., Logan, K., Horodyski, M., & Asif, I. M. (2018). Identifiable Factors Associated With Acceptance Into Sports Medicine Fellowship Programs. A Brief Report. *Clinical Journal of Sport Medicine*, *Publish Ahead of Print*. https://doi.org/10.1097/JSM.0000000000000694

Zhan, D., & Ward, A. R. (2019). Staffing, Routing, and Payment to Trade off Speed and Quality in Large Service Systems. *Operations Research*, *67*(6), 1738–1751. https://doi.org/10.1287/opre.2018.1838
