# Supplementary material for: Game-related assessments for personnel selection: A systematic review
Source: Front Psychol. 2022 Sep 28;13:952002. doi: 10.3389/fpsyg.2022.952002 (PMC9554090; doi:10.3389/fpsyg.2022.952002)
Supplement: Supplementary file 2 [file Presentation_2.pdf]

Supplementary Material - 2

Articles included in the systematic review

Albadán, J., Garcia Gaona, P. A., & Montenegro Marin, C. (2016). Assessment model in a selection process based in gamification. *IEEE Latin America Transactions*, *14*, 2789–2794. https://doi.org/10.1109/TLA.2016.7555256

Albadán, J., Gaona, P., Montenegro, C., González-Crespo, R., & Herrera-Viedma, E. (2018). Fuzzy Logic Models for Non-Programmed Decision-Making in Personnel Selection Processes Based on Gamification. *Informatica*, *29*, 1–20. https://doi.org/10.15388/Informatica.2018.155

al-Qallawi, S., & Raghavan, M. (2022). A review of online reactions to game-based assessment mobile applications. *International Journal of Selection and Assessment*, *30*, 14–26. https://doi.org/10.1111/ijsa.12346

Armstrong, M., Ferrell, J., Collmus, A., & Landers, R. (2016). Correcting Misconceptions About Gamification of Assessment: More Than SJTs and Badges. *Industrial and Organizational Psychology, 9*, 671-677. https://doi.org/10.1017/iop.2016.69

Auer, E. M., Mersy, G., Marin, S., Blaik, J., & Landers, R. N. (2022). Using machine learning to model trace behavioral data from a game-based assessment. *International Journal of Selection and Assessment*, *30*, 82–102. https://doi.org/10.1111/ijsa.12363

Balcerak, A., & Woźniak, J. (2021). Reactions to some ICT-based personnel selection tools. *Economics & Sociology*, *14*, 214–231. https://doi.org/10.14254/2071-789X.2021/14-1/14

Brown, M. I., Speer, A. B., Tenbrink, A. P., & Chabris, C. F. (2022). Using game-like animations of geometric shapes to simulate social interactions: An evaluation of group score differences. *International Journal of Selection and Assessment*, *30*, 167–181. https://doi.org/10.1111/ijsa.12375

Buil, I., Catalán, S., & Martínez, E. (2020). Understanding applicants' reactions to gamified recruitment. *Journal of Business Research*, *110*, 41–50. https://doi.org/10.1016/j.jbusres.2019.12.041

Collmus, A. B., & Landers, R. N. (2019). Game-Framing to Improve Applicant Perceptions of Cognitive Assessments. *Journal of Personnel Psychology*, *18*, 157–162. https://doi.org/10.1027/1866-5888/a000227

Egol, K. A., Schwarzkopf, R., Funge, J., Gray, J., Chabris, C., Jerde, T. E., & Strauss, E. J. (2017). Can video game dynamics identify orthopaedic surgery residents who will succeed in training? *International Journal of Medical Education*, *8*, 123–125. https://doi.org/10.5116/ijme.58e3.c236

Ellison, L. J., McClure Johnson, T., Tomczak, D., Siemsen, A., & Gonzalez, M. F. (2020). Game on! Exploring reactions to game-based selection assessments. *Journal of Managerial Psychology*, *35*, 241–254. https://doi.org/10.1108/JMP-09-2018-0414

Formica, E., Gaiffi, E., Magnani, M., Mancini, A., Scatolini, E., & Ulivieri, M. (2017). Can video games be an innovative tool to assess personality traits of the Millennial generation? An exploratory research. *Bollettino Di Psicologia Applicata*, *280*, 29–47.

Georgiou K. (2021). Can explanations improve applicant reactions towards gamified assessment methods? *International Journal of Selection and Assessment, 29*, 253–268. https://doi.org/10.1111/ijsa.12329

Georgiou, K., Gouras, A., & Nikolaou, I. (2019). Gamification in employee selection: The development of a gamified assessment. *International Journal of Selection and Assessment*, *27*, 91–103. https://doi.org/10.1111/ijsa.12240

Georgiou, K., & Lievens, F. (2022). Gamifying an assessment method: what signals are organizations sending to applicants? *Journal of Managerial Psychology, ahead-of-print*. https://doi.org/10.1108/JMP-12-2020-0653

Georgiou, K., & Nikolaou, I. (2020). Are applicants in favor of traditional or gamified assessment methods? Exploring applicant reactions towards a gamified selection method. *Computers in Human Behavior*, *109*, 106356. https://doi.org/10.1016/j.chb.2020.106356

Gkorezis, P., Georgiou, K., Nikolaou, I., & Kyriazati, A. (2021). Gamified or traditional situational judgement test? A moderated mediation model of recommendation intentions via organizational attractiveness. *European Journal of Work and Organizational Psychology*, *30*, 240–250. https://doi.org/10.1080/1359432X.2020.1746827

Harman, J. L., & Brown, K. D. (2022). Illustrating a narrative: A test of game elements in game-like personality assessment. *International Journal of Selection and Assessment*, *30*, 157–166. https://doi.org/10.1111/ijsa.12374

Hilliard, A., Kazim, E., Bitsakis, T., & Leutner, F. (2022). Measuring Personality through Images: Validating a Forced-Choice Image-Based Assessment of the Big Five Personality Traits. *Journal of Intelligence*, *10*, 12. https://doi.org/10.3390/jintelligence10010012

Hommel, B. E., Ruppel, R., & Zacher, H. (2022). Assessment of cognitive flexibility in personnel selection: Validity and acceptance of a gamified version of the Wisconsin Card Sorting Test. *International Journal of Selection and Assessment*, *30*, 126–144. https://doi.org/10.1111/ijsa.12362

Küpper, D. M., Klein, K., & Völckner, F. (2021). Gamifying employer branding: An integrating framework and research propositions for a new HRM approach in the digitized economy. *Human Resource Management Review*, *31*, 100686. https://doi.org/10.1016/j.hrmr.2019.04.002

Landers, R. N., & Collmus, A. B. (2022). Gamifying a personality measure by converting it into a story: Convergence, incremental prediction, faking, and reactions. *International Journal of Selection and Assessment*, *30*, 145–156. https://doi.org/10.1111/ijsa.12373

Landers, R. N., & Sanchez, D. R. (2022). Game-based, gamified, and gamefully designed assessments for employee selection: Definitions, distinctions, design, and validation. *International Journal of Selection and Assessment*, *30*, 1–13. https://doi.org/10.1111/ijsa.12376

Landers, R. N., Auer, E. M., & Abraham, J. D. (2020). Gamifying a situational judgment test with immersion and control game elements: Effects on applicant reactions and construct validity. *Journal of Managerial Psychology,35*, 225-239. https://doi.org/10.1108/JMP-10-2018-0446

Landers, R. N., Armstrong, M. B., Collmus, A. B., Mujcic, S., & Blaik, J. (2021). Theory-driven game-based assessment of general cognitive ability: Design theory, measurement, prediction of performance, and test fairness. *Journal of Applied Psychology*. https://doi.org/10.1037/apl0000954

Laumer, S., Eckhardt, A., & Weitzel, T. (2012). Online Gaming to Find a New Job – Examining Job Seekers' Intention to Use Serious Games as a Self-Assessment Tool. *German Journal of Human Resource Management: Zeitschrift Für Personalforschung*, *26*, 218–240. https://doi.org/10.1177/239700221202600302

McChesney, J., Campbell, C., Wang, J., & Foster, L. (2022). What is in a name? Effects of game-framing on perceptions of hiring organizations. *International Journal of Selection and Assessment*, *30*, 182–192. https://doi.org/10.1111/ijsa.12370

Melchers, K. G., & Basch, J. M. (2022). Fair play? Sex-, age-, and job-related correlates of performance in a computer-based simulation game. *International Journal of Selection and Assessment*, *30*, 48–61. https://doi.org/10.1111/ijsa.12337

Nikolaou, I. (2021). What is the Role of Technology in Recruitment and Selection? *The Spanish Journal of Psychology*, *24*, e2. https://doi.org/10.1017/SJP.2021.6

Nikolaou, I., Georgiou, K., & Kotsasarlidou, V. (2019). Exploring the Relationship of a Gamified Assessment with Performance. *The Spanish Journal of Psychology*, *22*, E6. https://doi.org/10.1017/sjp.2019.5

Sanchez, D. R., Weiner, E., & Van Zelderen, A. (2022). Virtual reality assessments (VRAs): Exploring the reliability and validity of evaluations in VR. *International Journal of Selection and Assessment*, *30*, 103–125. https://doi.org/10.1111/ijsa.12369

Wiernik, B. M., Raghavan, M., Caretta, T. R., & Coovert, M. D. (2022). Developing and validating a serious game-based assessment for cyber occupations in the US Air Force. *International Journal of Selection and Assessment*, *30*, 27–47. https://doi.org/10.1111/ijsa.12378

Woods, S. A., Ahmed, S., Nikolaou, I., Costa, A. C., & Anderson, N. R. (2020). Personnel selection in the digital age: A review of validity and applicant reactions, and future research challenges. *European Journal of Work and Organizational Psychology*, *29*, 64–77. https://doi.org/10.1080/1359432X.2019.1681401

Wu, F. Y., Mulfinger, E., Alexander, L., Sinclair, A. L., McCloy, R. A., & Oswald, F. L. (2022). Individual differences at play: An investigation into measuring Big Five personality facets

with game-based assessments. *International Journal of Selection and Assessment*, *30*, 62–81. https://doi.org/10.1111/ijsa.12360
