# Supplementary material for: Game-related assessments for personnel selection: A systematic review
Source: Front Psychol. 2022 Sep 28;13:952002. doi: 10.3389/fpsyg.2022.952002 (PMC9554090; doi:10.3389/fpsyg.2022.952002)
Supplement: Supplementary file 3 [file Presentation_3.pdf]

Supplementary Material - 3

Supplementary Table S1. Description of reviewed empirical studies

		Participants		GBA			
Study	Design	Size (%F)	Features	Name	Type	GBA measures	Other measures
Albadán et al. (2016)	CS	~50 (NR)	Professionals and university students	Unnamed	SGAD	Competences (dynamism, planning, integrity, self-control, conviction, perseverance)	-
Albadán et al. (2018)	CS	~50 (NR)	Professionals and university students	Unnamed	Otros/NA	Competences (dynamism, planning, integrity, self-control, conviction, perseverance)	-
al-Qallawi and Raghavan (2022)	CS	-	3146 user reviews	Thrive @ Sopra Steria<br>PwC Career Unlocked<br>Amazing Insights<br>Cosmic Cadet<br>KMPG Ready<br>Nautics<br>Skyrise City<br>Scoutible	Otros/NA	Not applicable	Sentiment<br>Emotions
Auer et al. (2022)	CS	621 (57.8%)	Undergraduate students (M_{age} = 23.34 years, 42.5% European American or white, 10.3% full-time employment)	Cognify	Otros/NA	Game performance<br>Game events<br>Mouse positions<br>Trace data modeling	CA - Fluid reasoning<br>CA - Processing speed<br>CA - Quantitative knowledge<br>CA - Verbal ability<br>CA - Virtual processing<br>Conscientiousness<br>GPA

		Participants		GBA			
Study	Design	Size (%F)	Features	Name	Type	GBA measures	Other measures
Balcerak and Woźniak (2021)	CS	833 (60.5%)	M_{age} = 32.6 years, 17.5% students	Not applicable	Otros/NA	Not applicable	Process favorability<br>Procedural justice
Brown et al. (2022)	CS	548 (82.0%)	Undergraduate students (38% White, 95% born in an English-speaking country, 94% identify as a native English speaker)	Shapes test	SGAG	Social intelligence	Social intelligence<br>Scholastic Aptitude Test / American College Testing
Buil et al. (2020)	CS	239 (31.0%)	M_{age} = 22.35 years	EY Challenge	SGAG	Game score	Perception of competence<br>Autonomy<br>Intrinsic motivation<br>Perceived ease of use<br>Perceived usefulness<br>Attitude<br>Satisfaction<br>Recommendation intention<br>Organizational attractiveness
Collmus and Landers (2019)	CS	659 (51.9%)	78.6% European American or White, 67.7% full-time employent	Not applicable	TA	Not applicable	CA - Three-dimensional rotation<br>CA - Letter and number series<br>CA - Matrix reasoning<br>CA - Progressive matrices<br>CA - Verbal reasoning<br>Time length<br>Applicant perceptions

Study	Design	Participants		GBA			Other measures
		Size (%F)	Features	Name	Type	GBA measures	
Egol et al. (2017)	CS	120 (NR)	-	Unnamed	SGAD	Heuristic flexibility<br>Social perception<br>Attentiveness<br>Processing speed<br>Memory<br>Adaptive and context dependent processing speed<br>Adaptive flexibility	Score in ABOS examination<br>Score in OITE examination<br>Score in USMLE examination
Ellison et al. (2020)	CS	347 (58.8%)	MTurk participants (M_{age} = 35.68 years, 63.5% Caucasian, 64.2% United States, 35.6% 2+ years of work experience)	gridChallenge<br>motionChallenge<br>digitChallenge<br>switchChallenge	SGAG<br>SGAG<br>SGAG<br>SGAG	Working memory<br>Complex planning capability<br>Basic numerical comprehension<br>Deductive logical reasoning test	Applicant reactions
Formica et al. (2017)	CS	981 (60.0%)	M_{age} = 23.0 years, 58% are gamers (62% casual gamers)	Not applicable	Otros/NA	Not applicable	Game mechanics checklist<br>Big Five personality

		Participants		GBA			
Study	Design	Size (%F)	Features	Name	Type	GBA measures	Other measures
Georgiou (2021)	EX	103 (55.3%)	M_{age} = 35.00 years, $M_{\text{job experience}}$ = 12.00 years, 42.8% had a master degree	Owiwi	SGAG	Competences (adaptability, resilience, flexibility, and decision-making)	Ease of faking<br>Opportunity to perform<br>Job-relatedness<br>Organizational attractiveness<br>Video game experience
	EX	186 (54.0%)	M_{age} = 30.04 years, $M_{\text{job experience}}$ = 6.93 years, 52.0% had a bachelor degree	Owiwi	SGAG	Competences (adaptability, resilience, flexibility, and decision-making)	Ease of faking<br>Opportunity to perform<br>Job-relatedness<br>Organizational attractiveness<br>Video game experience<br><br>Test-taking motivation<br>Recommendation intentions<br>Job acceptance intentions
Georgiou and Lievens (2022)	LO	100 (53.0%)	M_{age} = 31.77 years, $M_{\text{job experience}}$ = 8.21 years, 50% had a high school degree.	Owiwi	SGAG	Competences (adaptability, resilience, flexibility, and decision-making)	Perceived enjoyment<br>Flow<br>Organizational innovativeness and competence<br>Organizational attractiveness
	EX	103 (55.0%)	M_{age} = 35.88 years, $M_{\text{job experience}}$ = 12.00 years, 45% had a bachelor degree.	Owiwi	SGAG	Competences (adaptability, resilience, flexibility, and decision-making)	Perceived enjoyment<br>Flow<br>Organizational innovativeness and competence<br>Organizational attractiveness

Study	Design	Participants		GBA			Other measures
		Size (%F)	Features	Name	Type	GBA measures	
Georgiou and Nikolaou (2020)	EX	154 (45.5%)	Employees (M_{age} = 29.96 years, 47.7% university graduates)	Owiwi	SGAG	Competences (adaptability, resilience, flexibility, and decision-making)	Perceived fairness<br>Perceived predictive validity<br>Process satisfaction<br>Organizational attractiveness<br>Openness to experience
	CS	131 (65.0%)	University students and alumni (M_{age} = 26.0 years)				
Georgiou et al. (2019)	CS	20 (NR)	HR professionals	Owiwi	SGAG	Competences (adaptability, resilience, flexibility, and decision-making)	-
		321 (61.0%)	Business schools' students and graduates (M_{age} = 26.5 years)				
		410 (46.0%)	Job seekers (M_{age} = 29.0 years, 72% bachelor's or master's degree)				
Gkorezis et al. (2021)	EX	161 (44.1%)	Employees in a multinational information technology company	Owiwi	SGAG	Competences (adaptability, resilience, flexibility, and decision-making)	Organizational attractiveness<br>Recommendations intentions<br>Video game experience
Harman and Brown (2022)	EX	343 (58.0%)	75% White or Caucasian, 76.4% college education or more	GPM-nl<br>GPM-illustrated	SGAG<br>SGAG	Big Five personality	Big Five personality<br>Applicant reactions

		Participants		GBA			
Study	Design	Size (%F)	Features	Name	Type	GBA measures	Other measures
Hilliard et al. (2022)	CS	300 (69.0%)	Prolific Academic participants (M_{age} = 31.14 years)	Unnamed	SGAD	Big Five personality	Big Five personality
		431 (NR)	Prolific Academic participants (M_{age} = 40.0 years, 48.5% White)				
Hommel et al. (2022)	CS	180 (68.3%)	M_{age} =27.2 years, 80.6% students	Gamified Set-Shifting Task	SGAG	Cognitive flexibility	Cognitive flexibility<br>Cognitive ability<br>Acceptance of the assessment methods<br>Adaptability<br>Academic performance<br>Experience with original test<br>Computer experience<br>Online game experience
Landers and Collmus (2022)	CS	352 (79.0%)	Undergraduate psychology students (M_{age} =23.9 years, 49.6% White, 62.21% working at least part-time)	Unnamed	SGAG	Personality (conscientiousness and openness)	Personality (conscientiousness and openness)<br>Applicant reactions<br>Academic performance

		Participants		GBA			
Study	Design	Size (%F)	Features	Name	Type	GBA measures	Other measures
Landers et al. (2020)	EX	240 (43.8%)	MTurk participants (M_{age} =36.63 years, 86.6% were employed)	Unnamed situational-judgement test	SGAG	Self-management skills<br>Interpersonal skills	CA – Numerical reasoning<br>CA – Verbal reasoning<br>Personality (Conscientiousness)<br>Applicant reactions<br>Procedural justice<br>Distal organizational attitudes<br>Conscientiousness
Landers et al. (2021)	CS	633 (57.7%)<br><br>127 (59.3%)	Undergraduate students (M_{age} =21.35 years, 42.0% European American or White, 53.9% working at least part-time)<br><br>Employees (M_{age} =24.7 years, $M_{organizational\ tenure}$ =1.82 years)	Numbubbles<br>Resemble<br>Grid Lock<br>Proof It<br>Tally Up<br>Colour Pop<br>Short Cuts	SGAD<br>SGAD<br>SGAD<br>SGAD<br>SGAD<br>SGAD<br>SGAD	Cognitive ability	CA – Visual processing<br>CA – Fluid reasoning<br>CA – Processing speed<br>CA – Quantitative ability<br>CA – Verbal ability<br>Motivational outcomes<br>Attitudinal outcomes<br>Academic performance<br>Supervisor ratings
Laumer et al. (2012)	CS	1,882 (56.4%)	Jobseekers (37.8% students)	Not applicable	SGAG	Not applicable	Perceived selection fairness<br>Perceived privacy security<br>Perceived enjoyment<br>Perceived usefulness<br>Perceived ease of use<br>Intention to use self-assessments
McChesney et al. (2022)	CS	277 (NR)	MTurk participants (M_{age} =36.0 years, 65.0% Caucasian/White, 85% reported having looked for a job online at some point of their life)	Not applicable	TA	Not applicable	Perception of the organization (organizational innovation, organizational attraction)<br>Openness to experience<br>Propensity to innovation

		Participants		GBA			
Study	Design	Size (%F)	Features	Name	Type	GBA measures	Other measures
Melchers and Basch (2022)	CS	1,071 (32.6%)	Applicants (M_{age} =34.0 years)	Unnamed	SGAD	GBA score	Assessment center performance
Nikolaou et al. (2019)	CS	120 (64.0%)	University students and alumni (M_{age} =26.0 years, 55.0% currently employed)	Owiwi	SGAG	Competences (adaptability, resilience, flexibility, and decision-making)	Cognitive ability<br>Big Five personality<br>Overall job performance<br>Academic performance<br>Organizational citizenship behavior
Sanchez et al. (2022)	CS	112 (69.0%)	Undergraduate and graduate students (M_{age} =24.0, 38% Latino)	Project M	PG	Emotional intelligence	Video game pursuit<br>Intimidation with games<br>Emotional intelligence<br>Big Five personality
		100 (66.0%)	Undergraduate and graduate students (M_{age} =24.0, 38% Latino)	Richie's Plank Experience	PG	Sensation seeking<br>Heights aversion	Sensation seeking<br>Height aversion
		105 (79.0%)	University students (M_{age} =23.0, 35% Hispanic)	Balloon Pop	PG	Risk taking	Impulsiveness<br>Risk taking<br>Big Five personality

		Participants		GBA			
Study	Design	Size (%F)	Features	Name	Type	GBA measures	Other measures
Wiernik et al. (2022)	CS	466 (46.8%)	MTurk participants (74.5% White, 43.1% Bachelor's degree)	Virus Slayer	SGAD	Analytical thinking<br>Active learning<br>Deductive reasoning<br>Systems thinking<br>Adaptability<br>Situational awareness	-
		173 (39.3%)	MTurk participants (70.5% White, 52.0% has some college education)				
		156 (32.5%)	MTurk participants (48.0% Hispanic, 48.1% Bachelor's degree)				
Wu et al. (2022)	CS	565 (52.7%)	Psychology students (M_{age} =20.1, 33.1% White)	Click Town<br>Word Find	SGAD<br>SGAD	Conscientiousness (achievement striving, self-discipline, and cautiousness)	Big Five personality<br>Goal orientation<br>Cognitive ability<br>Game-play experience

Note. CS – Cross sectional study; EX – Experimental study; LO – Longitudinal study; NR – Non-reported; TA – Traditional assessment; SGAG – Serious game created by assessment gamification; SGAD –Serious game created by assessment gameful design; PG – Playful game; CA – Cognitive ability.
